# Expression and Subcellular Localization of Lanthipeptides in Human Cells

**DOI:** 10.1021/acssynbio.4c00178

**Published:** 2024-06-26

**Authors:** Sara M. Eslami, Chandrashekhar Padhi, Imran R. Rahman, Wilfred A. van der Donk

**Affiliations:** † Department of Chemistry and Howard Hughes Medical Institute, University of Illinois at Urbana−Champaign, Urbana, Illinois 61801, United States; ‡ Department of Biochemistry, University of Illinois at Urbana−Champaign, Urbana, Illinois 61801, United States

**Keywords:** cyclic peptides, cytolysin, haloduracin, organelle localization, protein−protein interactions, RiPPs

## Abstract

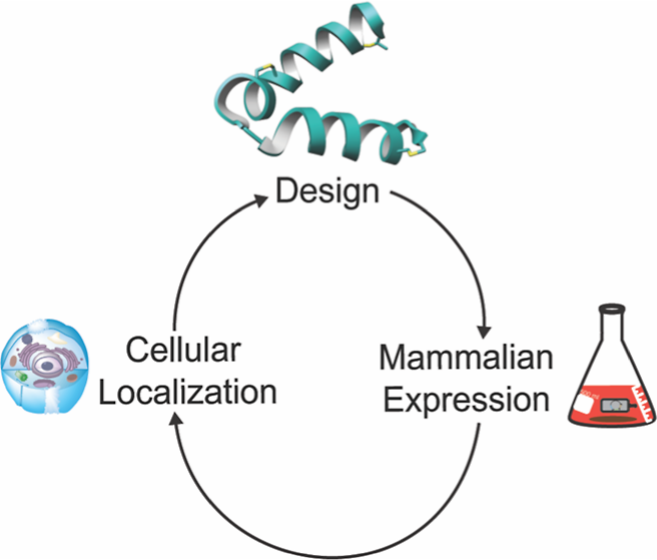

Cyclic peptides, such as most ribosomally synthesized and post-translationally modified peptides (RiPPs), represent a burgeoning area of interest in therapeutic and biotechnological research because of their conformational constraints and reduced susceptibility to proteolytic degradation compared to their linear counterparts. Herein, an expression system is reported that enables the production of structurally diverse lanthipeptides and derivatives in mammalian cells. Successful targeting of lanthipeptides to the nucleus, the endoplasmic reticulum, and the plasma membrane is demonstrated. In vivo expression and targeting of such peptides in mammalian cells may allow for screening of lanthipeptide-based cyclic peptide inhibitors of native, organelle-specific protein–protein interactions in mammalian systems.

## Introduction

Cyclic peptides represent a burgeoning area of interest in therapeutic and biotechnological research.^1−8^ Compared to their linear counterparts, cyclic peptides are more conformationally constrained and less susceptible to proteolytic degradation.^9^ Peptides contain a large surface area compared to small molecules, affording more opportunities to form points of contact for disruption of protein–protein interactions (PPIs). Cyclic peptides therefore present unique opportunities in the design of PPI inhibitors with enhanced or novel bioactivities.^4,6,10−13^ As the catalogue of cyclic ribosomally synthesized and post-translationally modified peptides (RiPPs) continues to grow,^14^ an ever-expanding area of research has focused on the biotechnological applications of RiPP structures and pathways.^14,15^

Current methods for the generation of cyclic peptide libraries that can be interrogated for target binding or disrupting PPIs include phage display, bacterial display, yeast display, mRNA display, split-intein circular ligation of peptides and proteins (SICLOPPS), and split-and-pool synthesis.^1,8,10,16−26^ Methods such as mRNA display and split-and-pool synthesis use in vitro screening. Conversely, in cellulo cyclization using SICLOPPS allows the identification of intracellularly functional inhibitors of PPIs using monocyclic peptides.^8,27^ Surface display technologies for enzymatically cyclized polycyclic peptides have been developed in bacteria,^17^ phage,^18,19^ and yeast^19^ and have typically been screened against extracellular targets. While a method for intracellular RiPP production combined with a bacterial reversed two-hybrid screen was successfully developed in *Escherichia coli*,^28^ the expression of enzymatically produced, multicyclic bacterial RiPPs has not been reported in mammalian cells.^29^ Such technology could be valuable because target proteins would be in their native environment in terms of PPIs and post-translational modifications.^8^

**Figure 1:**
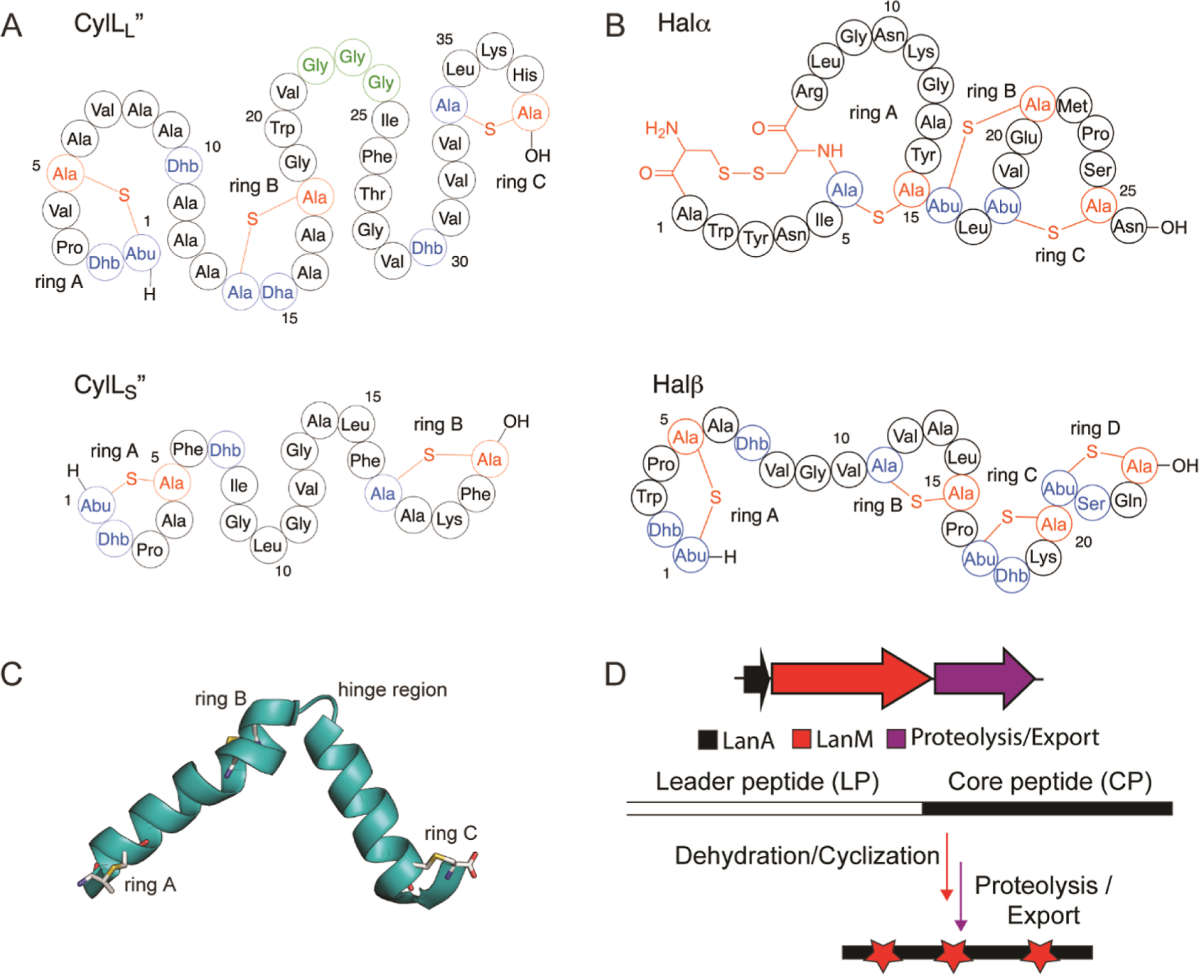
Schematics of the mature cytolysin and haloduracin ring patterns and their biosynthesis. (A) Structures of the two-component cytolysin composed of CylL_L_″ and CylL_S_″. Dehydrated residues are shown in blue, while former cysteine residues involved in ring formation are shown in red. The hinge region of CylL_L_″ is outlined in green. (B) Two-component lanthipeptide haloduracin α and β. (C) Ribbon diagram of one of the conformations observed in the NMR structure of the CylL_L_″ subunit in methanol (PDB ID: 6VGT)^39^ depicting two α-helices separated by the hinge region. Side chains of the residues participating in the rings are shown as sticks. (D) General biosynthetic machinery of class II lanthipeptides. Red stars represent thioether cross-links.

First identified in 1934, the two-component lanthipeptide cytolysin is a toxin produced by the Gram-positive bacterium *Enterococcus faecalis*, an opportunistic pathogen which, upon infection, may cause endocarditis, urinary tract infections, endophthalmitis, and alcoholic hepatitis.^30−32^ Isolation of the pAD1 plasmid encoding the enzymes for cytolysin biosynthesis revealed that two precursor peptides, CylL_L_ and CylL_S_, are modified into polycyclic products by a single LanM enzyme (CylM) and subsequently cleaved into their mature bioactive forms (CylL_L_″ and CylL_S_″) via the consecutive actions of the proteolytic activators CylB and CylA.^33,34^ CylL_L_″ and CylL_S_″ display potent synergistic activity against both bacteria and eukaryotic cells.^35^ Structural characterization showed that both peptides contain thioether cross-links, with CylL_L_″ containing three and CylL_S_″ containing two thioether rings (Figure 1A).^36,37^ With the successful production of cytolysin in an *E. coli* heterologous host, both CylL_L_″ and CylL_S_″ were demonstrated to be amenable to mutagenesis and structure–activity relationship (SAR) studies.^38^ Although cytolysin SAR has been explored, showing that the lanthipeptide synthetase CylM is tolerant toward changes in its substrate sequence, little has been done in pursuit of applying CylL_L_″ or CylL_S_″ for biotechnological applications. Given the covalently enforced structure of CylL_L_″ due to three thioether staples as determined by nuclear magnetic resonance (NMR) spectroscopy (Figure 1C),^39^ CylL_L_″ may be suitable as a template for disrupting PPIs at structurally complex interfaces.

Class II lanthipeptides are biosynthesized in bacteria as ribosomally synthesized precursor peptides (LanAs) composed of an N-terminal leader peptide that is important for recognition by the LanM lanthionine synthetases, and a C-terminal core peptide that is post-translationally modified to the final mature structure (Figure 1D).^40^ The LanM proteins first dehydrate Ser and Thr residues in the core peptide by phosphorylating the side chain alcohols, followed by phosphate elimination to form dehydroalanine (Dha) and dehydrobutyrine (Dhb), respectively.^41^ The same LanM protein then catalyzes Michael-type addition of the thiols of Cys residues to the Dha and Dhb residues to generate the characteristic thioether cross-links termed lanthionine (Lan) and methyllanthionine (MeLan), respectively.^42^ In the case of cytolysin, the substrate peptides (leader + core) are denoted CylL_L_ and CylL_S_, and the synthetase is CylM.^36^ The leader peptide can be removed with the protease CylA, resulting in the final products CylL_L_″ and CylL_S_″ (Figure 1A).^43^ In the case of haloduracin β, part of another class II two-component lanthipeptide (Figure 1B), the substrate is HalA2, and the lanthipeptide synthetase is HalM2.^44,45^ Proteolysis leads to the removal of the leader region, and the modified core peptide is termed Halβ (Figure 1B, bottom).

Herein, we describe the heterologous expression of CylL_L_ and HalA2 with their respective modification enzymes, CylM and HalM2, in mammalian cells, leading to the successful installation of multiple macrocyclic thioether linkages. The versatility of the CylL_L_ system in mammalian cells is demonstrated through variants of the core peptide undergoing modifications equivalent to those previously observed in the wild-type (WT) peptide. We also show successful localization of the lanthipeptides to the nucleus, endoplasmic reticulum (ER), and plasma membrane (PM), demonstrating the potential for organelle-targeted PPI disruption. Our work provides a method for cyclic peptide generation in mammalian cells, illustrating the applicability and utility of this system for potential future biotechnological applications such as transgenic antipathogen strategies in plants or antibody-like neutralization of pathogens or toxins in mammals.

## Results

### Design of a Lanthipeptide Mammalian Expression Vector

A multicistronic vector under the control of the constitutive CMV promoter was used for the expression of CylL_L_ (Figure S1). Because of differential codon usage and biases between bacteria and mammalian cells,^46^ the genes encoding the lanthipeptide synthetase CylM and its substrate peptide CylL_L_ were codon optimized for *Homo sapiens* expression and commercially synthesized (see Supporting Information for sequences). The genes encoding CylM and CylL_L_ were separated by the ribosomal-skipping, self-cleaving P2A peptide; P2A was chosen due to the observed higher expression of the second gene as well as the high self-cleavage efficiency of the peptide.^47−49^ To expand the versatility of the construct, we also included a FLAG tag and a Yellow Fluorescence-Activating and absorption-Shifting Tag (Y-FAST) at the N-terminus of the peptide. Y-FAST is a small protein, which fluoresces upon binding of its fluorogenic substrate.^50^ The FLAG-tag enables fixed cell immunofluorescence, and the Y-FAST provides the potential for dynamic and reversible live-cell imaging. The addition of the Y-FAST tag may also increase the normally short half-life of small peptides in the cell.^51^ Finally, a hexa-His tag was added to the N-terminus of the peptide to allow for purification via Ni^2+^ affinity chromatography. The construct was designed to test proof of principle and did not contain a selection marker; therefore, all data shown are from transient transfection. A second plasmid system was constructed with the same design but containing codon-optimized genes encoding HalA2 and HalM2.

### Expression of Lanthipeptides in Mammalian Cells

**Figure 2:**
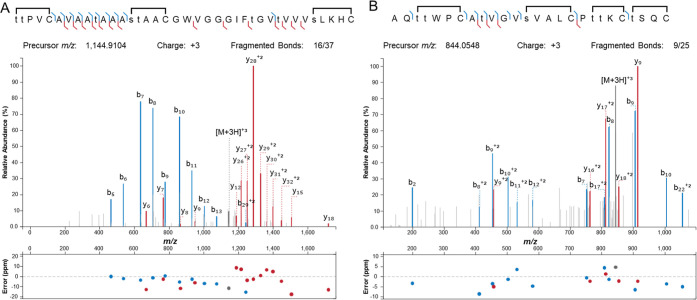
Electrospray ionization (ESI) high-resolution (HR)-MS/MS of lanthipeptides produced in Expi293F cells. (A) HR-MS/MS of CylA-digested CylL_L_″-S15T (8× dehydrated product) modified by CylM and (B) AspN-digested HalA2 (7× dehydrated product) modified by HalM2 in Expi293F cells. Dehydrated residues are indicated in lowercase. Brackets over the sequences represent the amino acid residues involved in macrocycle formation. Graph of the ppm errors for each identified ion is shown.^61^

CylL_L_″ requires the presence of the second cytolysin component, CylL_S_″, for cytolytic and antimicrobial activity. Additionally, the post-translationally modified CylL_L_ peptide requires the removal of its leader peptide to be activated and exhibit the aforementioned activity. Hence, we did not anticipate the production of modified CylL_L_, which still contains the leader peptide, to be toxic to the expressing cells. After coexpression of the His_6_-tagged precursor peptide and the CylM modification enzyme, the peptide was purified from the cell lysate by immobilized metal affinity chromatography (IMAC). Initial attempts at production of modified CylL_L_ in HEK293 cells resulted in an 8-fold dehydrated peptide. CylM-modified CylL_L_ produced in bacteria undergoes a maximum of seven dehydrations, with Thr27 escaping dehydration.^36^ Thus, in HEK293 cells, an extra loss of water is observed, leading to the dehydration of all Thr and Ser residues, possibly because of the design of the plasmid that produces more CylM compared to CylL_L_ in the mammalian cells than the bacterial cells. Additionally, modified CylL_L_ produced in HEK293 cells contained a glutathione (GSH) adduct (Figure S2). GSH adducts to dehydroamino acids (Dha/Dhb) have been observed previously during heterologous production of lanthipeptides in *E. coli*.^52−55^ In addition, mammalian LanC-like proteins (LanCL), which are present in HEK293 cells,^56^ are known to add GSH to dehydroamino acid residues and in vitro LanCL proteins added GSH to CylM-modified CylL_L_.^57^ To determine the location of the GSH adduct, a chymotrypsin digest was performed on modified CylL_L_ from HEK293 cells, followed by analysis by matrix-assisted laser desorption/ionization time-of-flight (MALDI-TOF) mass spectrometry (MS). The resultant 1719 Da fragment was analyzed by MALDI LIFT-TOF/TOF MS^58^ and was shown to correspond to the nonglutathionylated C-terminal portion of the core peptide (Figure S2). This observation indicated that the GSH adduct was located in the N-terminal portion of the core peptide. GSH adducts are more frequently observed on the more reactive Dha residue in comparison to Dhb, and therefore we hypothesized Dha15 to be glutathionylated. When Ser15 was mutated to Thr, and the variant CylL_L_-S15T peptide was coexpressed with CylM in a suspension cell line derivative of HEK293 (Expi293F), peptides underwent eight dehydrations, and no GSH adducts were observed (Figures 2 and S3A). Furthermore, assessment of potentially uncyclized Cys residues by derivatization with iodoacetamide (IAA)^59^ did not show any mass shift (+57.02 Da per alkylation of any uncyclized Cys thiol group) consistent with WT-like cyclization (Figure S3A). Correct cyclization was supported by the biological activity of the product after removal of the leader peptide with the protease CylA (vide infra) as well as the fragmentation patterns observed in tandem MS (Figure 2A). We hereafter refer to the peptide with eight dehydrations and the Dha15Dhb substitution after the removal of the leader peptide by CylA as CylL_L_″-S15T. For subsequent expressions, we utilized Expi293F because of the high level of peptide/protein expression reported for this cell line, owing to its ability to grow to relatively high-density cultures and the ease of scaling the expression volume.^60^

Following the successful production of CylL_L_″-S15T, we investigated if the methodology could also be applied to the production of another class II lanthipeptide, haloduracin β (Halβ). This peptide was chosen as previous research has shown high substrate tolerance of the modifying enzyme HalM2.^19,62−64^ Similar to the results with CylL_L_, the precursor HalA2 expressed together with the synthetase HalM2 in Expi293F cells underwent up to seven dehydrations, with tandem MS demonstrating that all Thr and Ser residues were dehydrated except Ser22 (when counted from the starting residue of the core peptide, Figure S3D) escaping dehydration (Figure 2B); this residue is also not dehydrated in native Halβ. Cyclization of HalA2 was investigated by reaction with *N*-ethylmaleimide (NEM)^65^ instead of the IAA that was used for CylL_L_, as NEM was used in a previous study on HalA2 modification.^66^ Incubation of HalM2-modified HalA2 revealed no NEM adducts (+125.05 Da, typical of Cys-free thiol derivatization with NEM), indicating complete cyclization (Figure S3B).

### Bioactivity of Lanthipeptides Produced in Mammalian Cells

**Figure 3:**
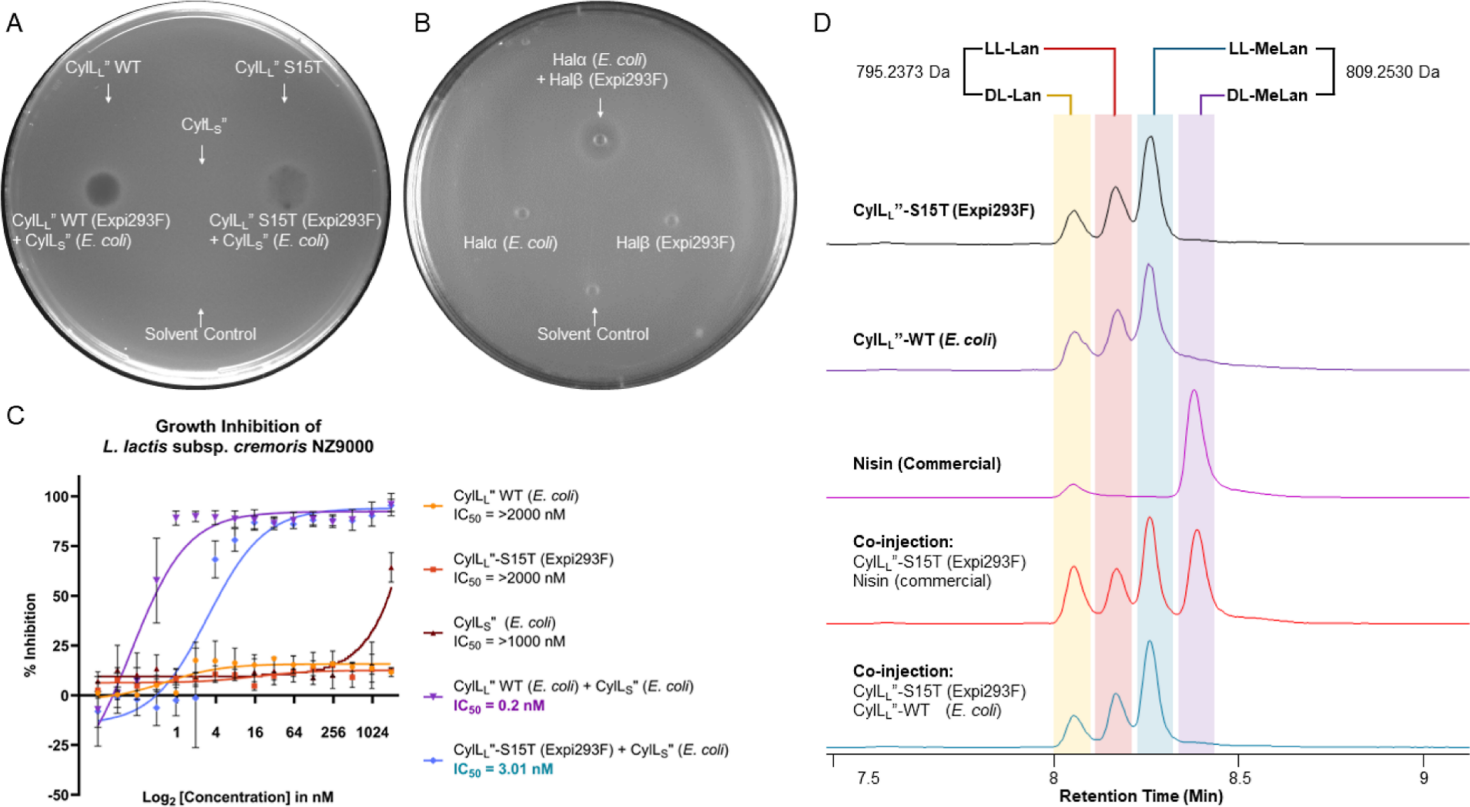
Bioactivity of cytolysin and haloduracin produced in mammalian cells. (A) Synergistic activity of CylL_L_″ produced in Expi293F cells and CylL_S_″ produced in *E. coli* against *L. lactis* sp. cremoris NZ9000. CylL_L_″ wild-type (WT) by itself does not inhibit the growth of the test bacteria but shows a clear zone of inhibition when spotted along with CylL_S_″. CylL_L_″-S15T shows a similar activity profile; however, the extent of bacterial growth inhibition was lower than that for WT. CylL_S_″ by itself also does not show any noticeable inhibition of the indicator bacteria. Solvent control of 20% methanol in PBS was used. (B) A similar approach was applied for investigating the synergistic activity of Halβ produced in Expi293F cells in combination with Halα produced from *E. coli*. The indicator strain tested in this case was *L. lactis* CNRZ 117. Halβ contained two amino acids from the leader peptide (Ala-Gln) at its N-terminus (see text). For (A,B), each peptide was tested at 100 pmol individually or combined with its partner peptide against the indicator strains. (C) Quantitative bacterial inhibition assays for CylL_L_″-S15T produced in Expi293F cells and CylL_L_″ WT obtained from *E. coli*, individually or in combination with CylL_S_″ produced in *E. coli*, against *L. lactis* sp. cremoris NZ9000 plotted as a nonlinear regression curve. IC_50_ values are enumerated in the graph legend. (D) Stereochemistry of the 8-fold dehydrated CylL_L_″-S15T (Expi293F) product determined by advanced Marfey’s analysis using CylL_L_″ WT (*E. coli*) and nisin (*L. lactis*; Sigma) as standards. Co-injections of the standards and the test peptide are shown. Extracted ion chromatograms for derivatized diastereomers of lanthionine (LL- or DL-Lan) at [M – H] = 795.2373 Da and methyllanthionine (LL- or DL-MeLan) at [M – H] = 809.2530 Da are shown in colored highlights. Derivatized MeLan ionizes better than Lan.

To confirm the formation of the native ring patterns in modified CylL_L_-S15T and HalA2 generated in Expi293F cells, we tested the respective modified core peptides for antimicrobial activity after proteolytic digestion by expressed and purified CylA and commercial AspN, respectively. WT CylL_L_″ (but with 8-dehydrations) produced in Expi293F cells when combined with *E. coli*-expressed CylL_S_″^36^ demonstrated synergistic antimicrobial activity toward *Lactococcus lactis* sp. cremoris NZ9000, as demonstrated by a clear zone of growth inhibition (Figure 3A). CylL_L_″-S15T from Expi293F cells combined with bacterially produced CylL_S_″ also exhibited inhibitory activity (Figure 3A). Quantification of the antimicrobial activity in liquid media (Figure 3C) showed that *E. coli* produced WT CylL_L_″ in combination with CylL_S_″ from *E. coli* exhibited similar antibiotic efficacy as reported in past studies^38^ (IC_50_ = 0.2 nM), whereas CylL_L_″-S15T when combined with CylL_S_″ from *E. coli* displayed clear synergistic activity but with ∼15-fold lower potency (IC_50_ = 3.0 nM). WT CylL_L_″ (*E. coli*) and CylL_L_″-S15T (Expi293F) by themselves did not exhibit any growth inhibition of bacteria up to the tested concentrations (Figure 3C). The reduction in activity of the Expi293F-produced S15T mutant of CylL_L_″ is consistent with a previous study that reported that the mutation of Ser15 to Ala led to ∼8-fold attenuated antimicrobial activity.^38^ The reduced activity observed in this study is a combination of the S15T mutation and the fact that the CylL_L_″ made in Expi293F cells is dehydrated 8-fold, whereas the product in *E. coli* is dehydrated seven times.

Endoproteinase AspN-cleavage of HalM2-modified HalA2 should lead to the release of the core peptide with five extra amino acid residues (Asp-Val-His-Ala-Gln) at the N-terminus resulting from the leader peptide (Figure S3C). Previous studies have shown that up to six extra residues at the N-terminus of Halβ did not attenuate synergistic bioactivity with Halα.^44^ Surprisingly, AspN cleavage of HalA2 modified by HalM2 in Expi293F cells resulted in Halβ with only two amino acid residues from the leader peptide (Figure 2B), presumably due to amino peptidase activity in either the sample or commercial AspN. We assayed this material for growth inhibitory activity against the indicator strain *L. lactis* CNRZ 117 separately as well as in combination with Halα (Figure 1C) produced in *E. coli*.^67^ The α and β subunits alone did not inhibit the growth of *L. lactis*, but when combined, we observed a zone of growth inhibition demonstrating synergistic activity and thus supporting the formation of the native ring pattern of haloduracin β produced in Expi293F cells (Figure 3B).

### Stereochemistry of Cytolysin Component Produced in Mammalian Cells

To provide further evidence that the CylL_L_″-S15T variant produced in Expi293F cells was the result of correct enzymatic modification, we determined the stereochemistry of the Lan and MeLan residues in CylL_L_″-S15T. We used WT CylL_L_″ obtained from *E. coli* and commercially available nisin from *L. lactis* as standards. WT CylL_L_″ contains one LL-MeLan, one LL-Lan, and one DL-Lan,^36,37^ whereas nisin contains one DL-Lan and four DL-MeLan.^68^ Bis-derivatization of Lan/MeLan residues from a hydrolyzed lanthipeptide using the advanced Marfey’s reagent *N*^α^-(5-fluoro-2,4-dinitrophenyl)-l-leucinamide (L-FDLA) leads to diastereomers with masses [M – H] = 795.2373 Da (for Lan) and [M – H] = 809.2530 Da (for MeLan).^69^ The extracted ion chromatogram of CylL_L_″-S15T produced in Expi293F cells was near identical to that of WT CylL_L_″ produced in *E. coli*, showing LL-Lan, DL-Lan, and LL-MeLan, whereas no DL-MeLan was observed (Figure 3D). This observation, in combination with the tandem MS data (Figure 2A), strongly suggests that the CylL_L_″-S15T variant has the same stereochemistry and cyclization patterns as the WT CylL_L_″.

### CylL_L_ Mutant Peptides Are Expressed and Modified in Mammalian Cells

**Figure 4:**
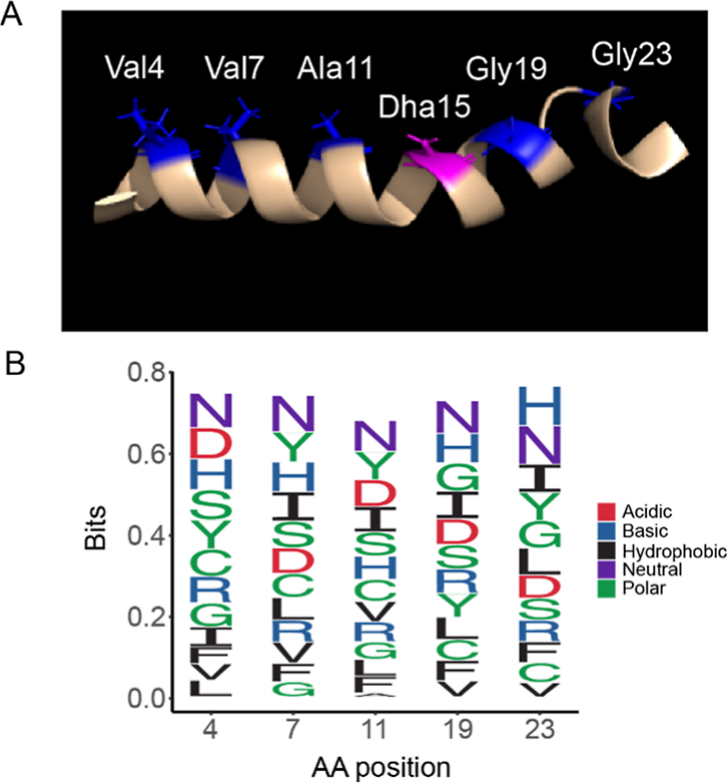
Design of the NDT mutant library of CylL_L_″. (A) NMR structure of CylL_L_″ (PDB ID: 6VGT; BMRB ID: 30710)^39^ dissolved in methanol, showing mutated residues in blue and the position of the S15T substitution for CylL_L_-S15T in pink as Dha15. (B) Frequency of NDT-encoded amino acids at each mutation site in the sequenced library. Sequence logo was generated in RStudio (V1.3.959) using the ggplot2 and ggseqlogo packages.^70^

CylL_L_-S15T was used as a template to generate a mutant library using the NDT degenerate codon (D = A/G/T). The five mutated positions were chosen along one helical face spanning the A and B rings of CylL_L_-S15T based on its NMR structure (Figure 4A). Previous lanthipeptide libraries have employed NNK, NWY, and NDT degenerate codons.^19,28,62^ For this study, NDT codons were chosen for the following reasons. First, they lead to a diverse array of amino acids, including both positively and negatively charged residues (Gly, Val, Leu, Ile, Cys, Ser, Arg, His, Asp, Asn, Phe, and Tyr). Second, no stop codons are introduced, and third, each codon encodes one amino acid, thereby preventing overrepresentation of a single amino acid.

**Figure 5:**
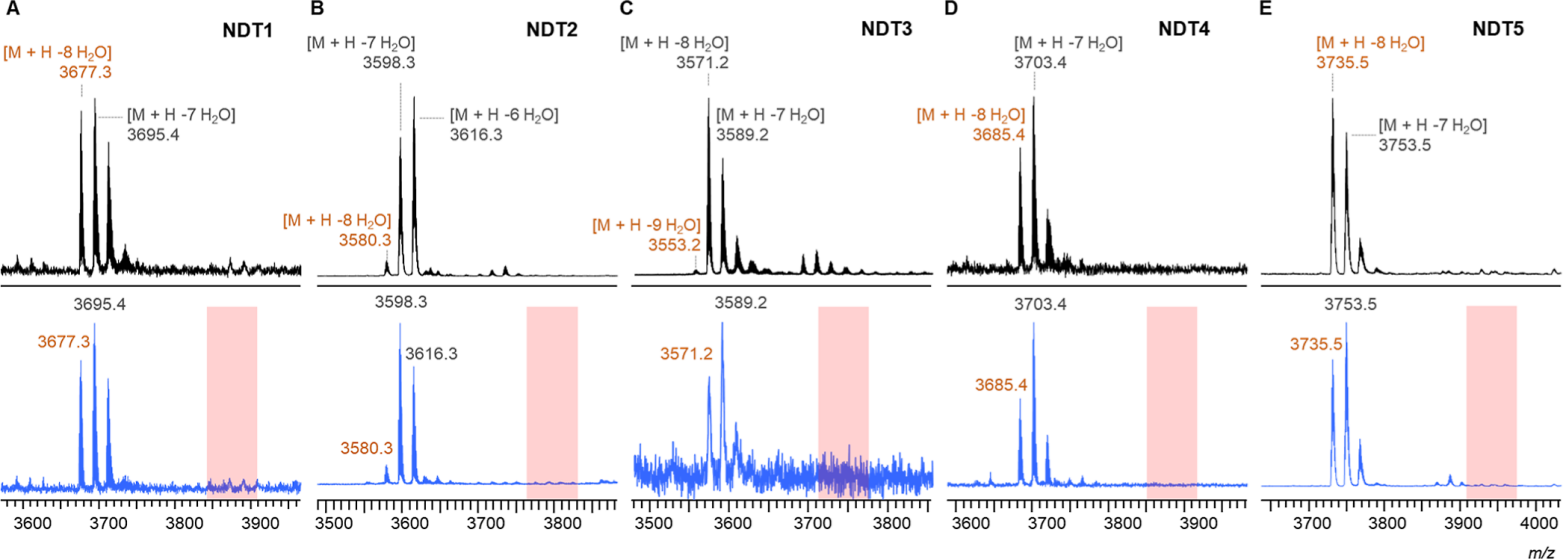
MALDI-TOF MS of CylA-digested CylL_L_-S15T NDT variants modified in Expi293F cells by CylM before (black) and after (blue) iodoacetamide (IAA) reaction. Average isotopic mass for the products with the highest number of dehydrations observed in each mutant peptide is shown in orange font. In the case of NDT3, the 9× dehydrated product was not observed after the IAA reaction, probably due to its low abundance. Peptides were expressed in Expi293F cells, purified via Ni-NTA chromatography, digested with CylA, and analyzed by MALDI-TOF MS before and after IAA alkylation. The rectangles in red encompass the mass range where alkylated peptides would be detected if alkylation products were formed. No peaks in the red zones suggested the formation of all three rings (no free Cys thiols) in the NDT mutants, a conclusion supported by the HR-MS/MS data in Figures S4–S8.

Deep sequencing revealed a library size of 1.74 × 10^5^ unique sequences, representing ∼70% coverage of the theoretical library size (12^5^ = 2.49 × 10^5^, for 12 residues at 5 positions). The incomplete coverage may be explained by the observed transfection efficiency as well as the observation of a slight overrepresentation of the template sequence. Generally, however, the distribution of amino acids at the variable positions was near the statistical prediction (Figure 4B). To determine if CylM could successfully modify CylL_L_-S15T derivatives in mammalian cells, five sequences from the NDT library (NDT1–5) were chosen randomly for expression in Expi293F cells. All five sequences were modified with up to eight dehydrations (nine in the case of NDT3) after coexpression with CylM (Table 1). Dehydrations of Ser/Thr residues were confirmed via high-resolution tandem MS (HR-MS/MS), and cyclization was confirmed via IAA treatment, as discussed in previous sections (Figure 5). Additionally, HR-MS/MS data for all five variants supported similar ring patterns as that of WT CylL_L_″ (Figures S4–S8). Mutation of a hinge region residue (Gly23) to Ser in variant 2 did not affect the ring pattern, and no dehydration was observed of Ser23 (Figures 5B and S5). However, mutation of Val7 in the N-terminal helix region to Ser in the NDT3 variant resulted in a very small amount of 9× dehydrated product in which the introduced Ser was dehydrated (Figure 5C).

**Table 1:**
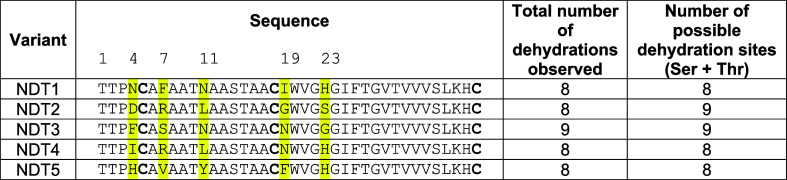
CylL_L_″-S15T NDT Variants^a^

a Residues highlighted in yellow depict the mutated amino acids. Residue numbering of these residues is shown.

### Lanthipeptides Can be Targeted to Organelles in Mammalian Cells

**Figure 6:**
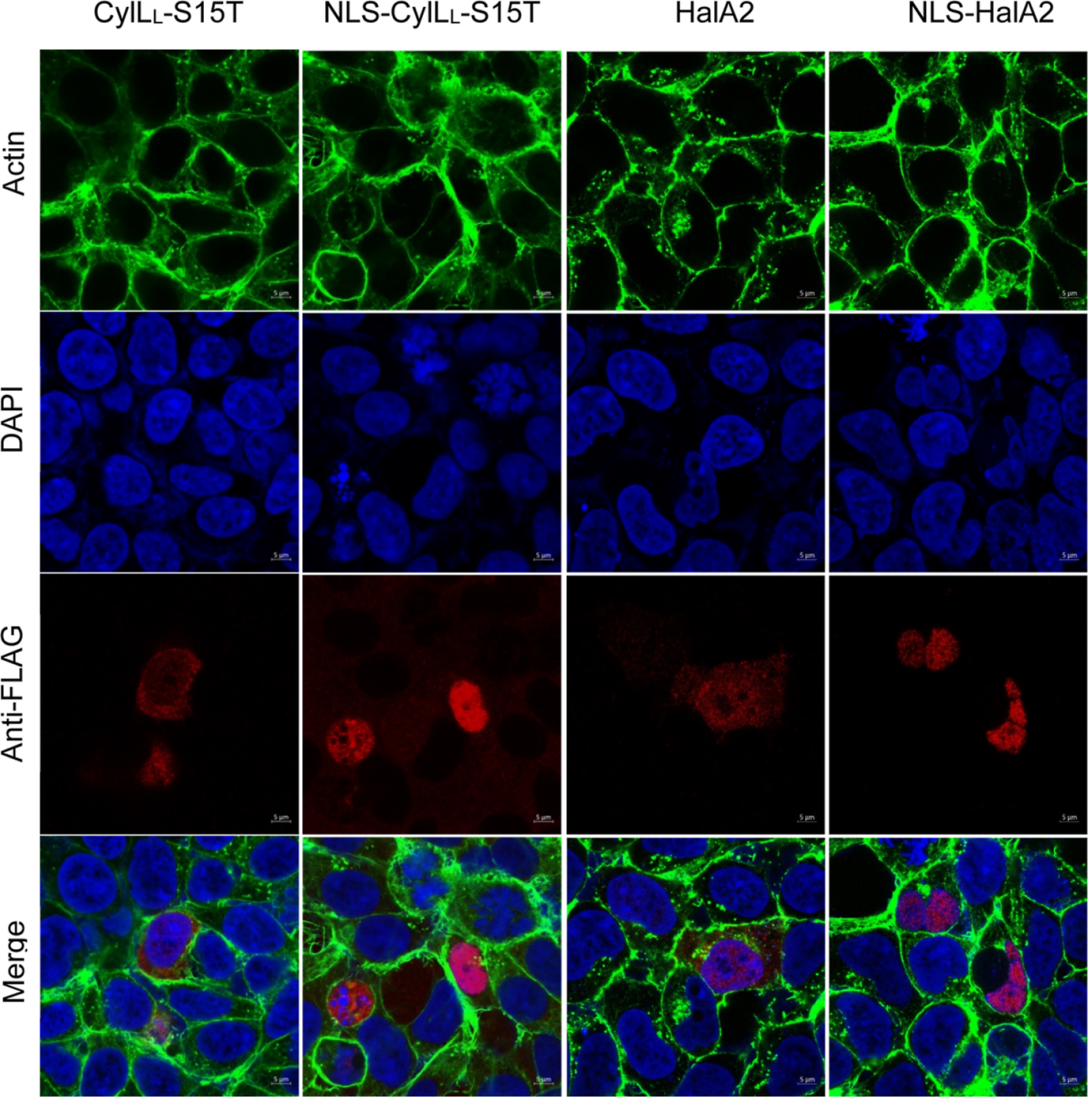
Confocal micrographs of untargeted and nuclear-targeted CylL_L_-S15T and HalA2 coexpressed with CylM and HalM2, respectively. HEK293 cells were fixed and permeabilized 2 days post-transfection. The following stains were used. DAPI (blue, nucleus), phalloidin-488 (green, actin), and mouse anti-FLAG primary antibody with goat antimouse Fluor647 secondary antibody (red, CylL_L_-S15T). Immunofluorescence was visualized at 63× magnification using an LSM880 confocal microscope.

Given the successful modification of HalA2, CylL_L_-S15T, and the NDT variants, we next focused on targeted localization of modified CylL_L_-S15T and HalA2 within mammalian cells. Nuclear targeting was chosen first because many transcription factor interactions in the nucleus may be targets of PPI inhibition. A nuclear localization signal (KKKRKV) was appended to the N-terminus of FLAG-tagged precursor peptides. Immunostaining of transfected HEK293 cells with an anti-FLAG antibody demonstrated colocalization of CylL_L_-S15T and HalA2 with the nuclear counterstain DAPI compared to the untargeted controls (Figure 6). To confirm that the nuclear-targeted lanthipeptides were still fully modified, nuclear-localized CylL_L_-S15T was expressed in Expi293F cells with nontargeted CylM, and subsequent IMAC purification confirmed a dehydration profile (up to 8× dehydrations) similar to the nonlocalized CylM-modified CylL_L_-S15T produced in Expi293F cells (Figure S9). Thus, CylM was able to modify CylL_L_ prior to transport to the nucleus. An equivalent outcome was observed for nuclear-localized HalA2 that also underwent up 7× dehydrations (Figure S9).

**Figure 7:**
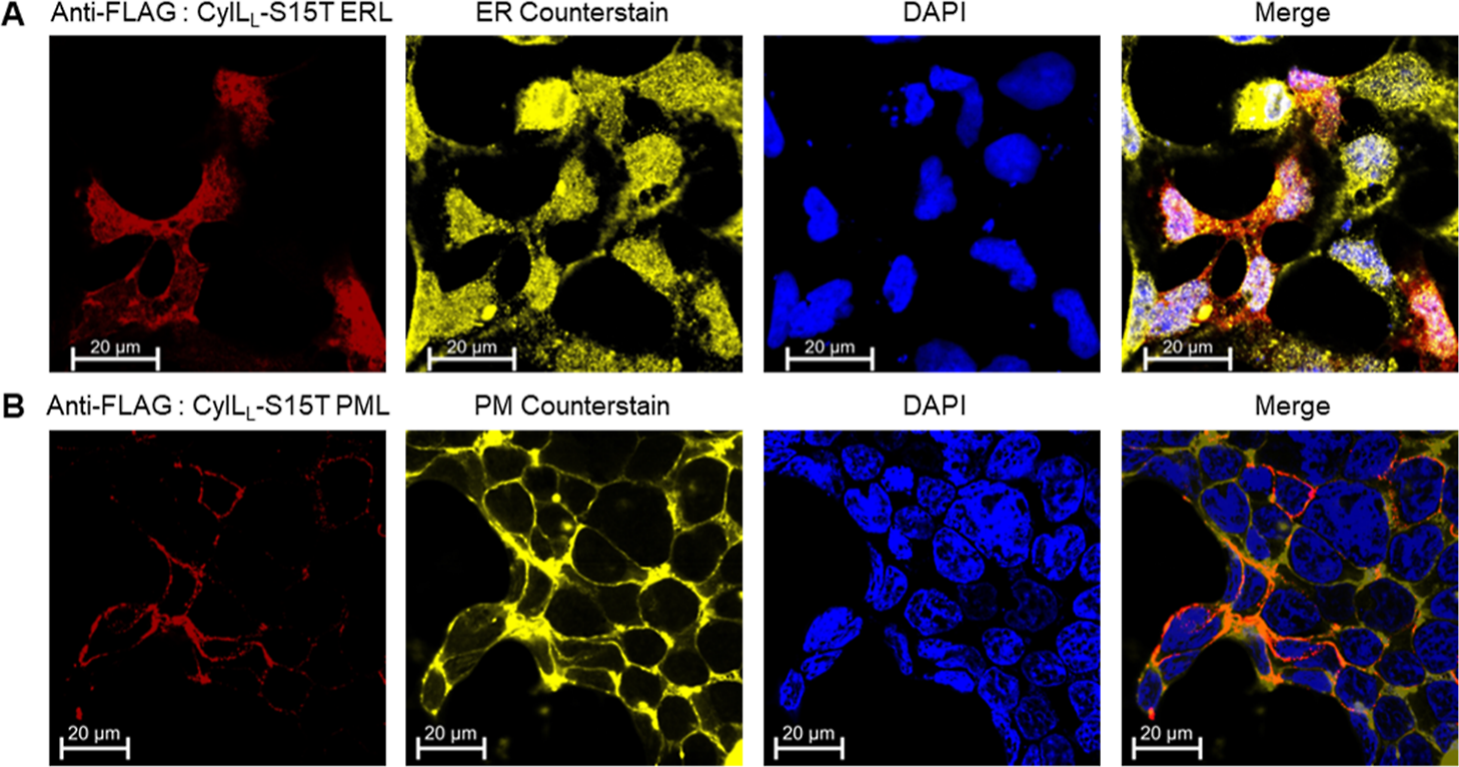
Confocal micrographs of endoplasmic reticulum localization (ERL) and plasma membrane localization (PML)-targeted CylL_L_-S15T coexpressed with CylM. HEK293 cells were transfected with the appropriate expression vector, fixed and permeabilized 2 days post-transfection. In all cases, the following stains were used. DAPI (blue, nucleus) and mouse anti-FLAG primary antibody with goat antimouse Fluor647 (λ_ex_ = 647 nm) secondary antibody (red, CylL_L_-S15T). (A) For the ERL multiplexing experiment, ER-specific anti-GRP94 antibody (raised in rabbit) was used, which was targeted with goat antirabbit Fluor555 (λ_ex_ = 568 nm) secondary antibody (yellow). (B) For PML, a PM counterstain, CellBrite Orange cytoplasmic membrane dye (λ_ex_ = 568 nm; pseudo color processed in yellow) was used as per the manufacturer’s instructions. In all cases, immunofluorescence was visualized at 63× magnification using an LSM900 confocal microscope. Data analysis, micrograph presentation, and scaling were performed using ZEISS ZEN lite v3.9.

A similar approach was implemented for CylL_L_-S15T localization to the ER by extending its C-terminus by a KDEL localization motif. Since the C-ring in CylL_L_-S15T is formed at the ultimate residue of the resultant peptide, a GAG-linker was inserted between the peptide and the KDEL-receptor recognition sequence (Figure S10A,C). This construct indeed resulted in localization of the peptide to the ER in transfected HEK293 cells (Figure 7A). Additionally, we also show that extension of the CylL_L_-S15T core region by attachment of the GAG-linker, and the KDEL signaling sequence did not affect the ability or efficiency of CylM in introducing the expected modifications in the peptide (Figure S11A,C).

Targeting of CylL_L_-S15T to the PM by myristoylation required construct-refactoring since ribosomal skipping and self-cleaving at the P2A site with the existing plasmid architecture would have led to an N-terminal proline residue. An N-terminal glycine residue is a prerequisite for host *N*-myristoyltransferases to catalyze lipidation for membrane incorporation.^71^ Therefore, the gene encoding the CylL_L_-S15T (fused to FLAG-6xHis-YFast as shown in Figure S10B,D) was refactored after the CMV promoter, followed by the P2A site and the gene of the CylM maturase. The PM localization signal (PML) GCIKSKRKDG^72^ was incorporated on the N-terminus of the peptide. This design successfully localized the peptide to the PM in transfected HEK293 cells (Figure 7B). The resultant peptide remained 8× dehydrated (Figure S11B,D). This observation further demonstrates the amenability of the system and the potential for generating variant libraries for disrupting PPIs in a native environment.

## Discussion

Herein, we describe the production of polycyclic lanthipeptides in mammalian cells as well as the successful localization of these peptides to various organelles. Owing to their stability and structural diversity, cyclic peptides have garnered much interest for their therapeutic potential.^4,73^ Previous research has employed display and screening technologies to identify enzymatically generated polycyclic PPI inhibitors in bacteria and yeast.^29^ Expression of such peptides in mammalian cells may allow for functional screening against native PPIs.

Disrupting protein secondary structures can be accomplished through small molecules, antibodies, or peptides. Small molecule compounds cannot easily mimic the extended, globular three-dimensional interfaces and also face high rates of clearance; antibodies, due to their large size, are mainly employed in disrupting extracellular PPIs.^74^ Cyclic peptides may fill the gap because of their secondary structures, comparatively small sizes, and resistance to proteolytic cleavage. Our results demonstrate the utility of lanthipeptide biosynthetic enzymes in the production of a diverse set of polycyclic structures within mammalian cells. Consequently, this method may expand the scope of therapeutic targets accessible to enzymatically generated polycyclic peptides.

## Materials and Methods

### Buffers and Media

LanA resuspension buffer (B1) contained 6.0 M guanidine hydrochloride, 0.5 mM imidazole, 500 mM NaCl, and 20 mM NaH_2_PO_4_ in deionized (DI) H_2_O, and the pH was adjusted to pH 7.5. LanA wash buffer (B2) contained 20 mM NaH_2_PO_4_, 30 mM imidazole, and 300 mM NaCl in DI H_2_O, and the pH was adjusted to 7.5. LanA elution buffer (EB) contained 20 mM Tris HCl, 1.0 M imidazole, and 100 mM NaCl in DI H_2_O, and the pH was adjusted to 7.5. LanM start buffer contained 20 mM Tris HCl, 1 M NaCl, and pH 7.6, and LanM final buffer was made up of 20 mM HEPES, 300 mM KCl, and pH 7.5. For NDT mutants, wash buffer constituted 50 mM Tris HCl, 40 mM imidazole, and 300 mM NaCl adjusted to pH 8. The elution buffer used was of similar constituency, except the concentration of imidazole was 500 mM. For buffer exchange, a solution of 50 mM Tris HCl supplemented with 100 mM NaCl was used. A 1× solution of Dulbecco’s phosphate buffered saline (PBS, pH 7.4) was used. The pH of all buffers was adjusted using 1 or 5 M NaOH and HCl and passed through a 0.2 μm filtration device.

### Expression and Purification of Protease CylA

A culture of 10 mL LB with 10 μL of kanamycin solution (50 mg/mL; Kan50) was inoculated with *E. coli* BL21 cells containing pRSF_His-CylA^43^ from a frozen stock and incubated overnight at 37 °C, with shaking. A 1 L culture of Terrific Broth (TB) with 1 mL of Kan50 was inoculated with 10 mL of the overnight culture and incubated at 37 °C, with shaking at 220 rpm. The culture was induced with 500 μM IPTG (1 M stock) at mid log phase and shaken overnight at 18 °C at 200 rpm. The culture was pelleted at 4500*g* for 15 min, and the resulting pellet was resuspended in 40 mL of LanM start buffer with lysozyme and allowed to incubate for 20 min on ice. Lysis was performed via sonication (39% amplitude, 4.4 s on, 9.9 s off) for at least 5 min. The lysate was pelleted at 13,000*g* for 30 min and filtered through a 0.45 μm filter. Purification was performed using IMAC with a 5 mL HisTrap column and an AKTA FPLC. The following conditions were used: the column was equilibrated with 2–3 column volumes (CV) of LanM start buffer, followed by loading of the lysate; buffer A = LanA B2 and buffer B = elution buffer. The column was washed with 10% B for 2 CV, 15% B for 4 CV, and 50% B for 4 CV. Fractions containing CylA were determined via SDS-PAGE with a 4–20% Tris gel at 200 V and collected, concentrated, and the buffer exchanged into LanM final buffer using a 30 kDa MWCO amicon filter. Aliquots were stored in LanM final buffer with 10% glycerol at −70 °C.

### Expression and Purification of CylL_L_ in HEK293 Cells

The plasmid construct encoding CylM and CylL_L_ under a constitutive promoter was used to transfect HEK293 cells using Turbofect transfection reagent (Thermo Fisher). HEK293 cells were grown in a 100 mm culture dish and were 70–90% confluent at the time of transfection. Cell media was changed 3–4 h post-transfection, followed by an overnight incubation at 37 °C, 5% CO_2_. After incubation, media was aspirated, and the cells were washed with cold 1× PBS. After PBS removal, 5 mL of Pierce IP lysis buffer and 1/2 of a Pierce protease inhibitor tablet were added to each 100 mm dish and incubated for 5–10 min at 4 °C. The cell lysate was pelleted at 1000*g* for 5 min, and the supernatant was purified via Ni resin affinity chromatography. The supernatant was washed with 3 CV of LanM start buffer with 25 mM imidazole, and the peptide was eluted in 1 CV of LanM start buffer with 0.5 M imidazole. The elution was treated with 5–10 μL of 1.6 μg/μL CylA and allowed to incubate overnight at RT. After acidification with 50% TFA/H_2_O, the elution was filtered through a 0.45 μm filter and purified on an Agilent 1260 Infinity analytical HPLC (aHPLC) with a Luna C8 (100 A) analytical column. The following conditions were used with a flow rate of 0.80 mL/min with solvent A containing H_2_O + 0.1% TFA and solvent B containing ACN + 0.1% TFA: 2% B for 5 min, 2% B – 100% B over 20 min, and 100% B for 5 min. UV absorbance was monitored at 220 and 254 nm. Fractions were collected manually and analyzed via MALDI TOF MS and LIFT fragmentation using a Bruker UltrafleXtreme mass spectrometer.

### Generation of CylL_L_-S15T

Site-directed mutagenesis was performed using overlapping primers. The following polymerase chain reaction (PCR) conditions were used with Phusion polymerase in a 50 μL reaction volume. Each reaction contained 1× Phusion HF buffer, 1 M betaine, 0.2 mM dNTPs, 0.5 μM of each primer, 0.5 μL polymerase, at least 10 ng of DNA template, and DI H_2_O added up to 50 μL. Initial denaturation was performed for 2 min at 95 °C, followed by 26 cycles of denaturation (20 s, 95 °C), annealing (30 s, 69.8 °C), and elongation (7 min, 72 °C), and a final elongation step (72 °C) for 10 min. Successful amplification was confirmed via gel electrophoresis. After amplification, 1 μL of Dpn1 enzyme was added to each 50 μL reaction and incubated at 37 °C for 1 h, followed by a Qiagen PCR cleanup per the manufacturer’s instructions. Subsequently, 5 μL of amplified product was added to 50 μL of chemically competent NEBTurbo cells. Cells were kept on ice for 30 min, followed by heat shock at 42 °C for 30 s and recovery with 1 mL LB for 1 h. After recovery, cells were pelleted, and ∼700 μL of supernatant was removed; the pellet was resuspended in the remaining liquid, of which 50 μL was plated onto LB/Amp100. Following overnight incubation at 37 °C, single colonies were used to inoculate 10 mL LB/Amp100 and grown overnight at 37 °C. Plasmid extraction (Qiagen miniprep) was performed using the overnight cultures, and plasmids were sequenced to determine successful mutagenesis.

### Expression and Purification of Lanthipeptides in Expi293F Cells

Expi293F cells were transfected with expression plasmids using the ExpiFectamine 293 Transfection Kit (Gibco) per the manufacturer’s instructions. Expression enhancers 1 and 2 were added, per the manufacturer’s instructions, 19–21 h post-transfection, followed by 4 days of incubation at 37 °C, 8% CO_2_. Cells were pelleted at 1000*g* for 5 min, and the resulting supernatant was removed. For the purification of CylL_L_-S15T and HalA2, 3–5 mL of Pierce IP lysis buffer was added to the cell pellet, and the sample was incubated with gentle agitation at RT for 5 min. The lysate was pelleted at 1000*g* for 5 min, and the supernatant was purified via nickel(II)-nitrilotriacetic acid (Ni-NTA) affinity chromatography and analytical HPLC, as described above. In the case of the NDT mutants, ER-targeted and PML-targeted CylL_L_-S15T production discussed in the sections below, cells were allowed to grow for 5 days after transfection with the appropriate plasmid construct, followed by harvesting of the cells by centrifugation at 1000*g* for 10 min. The spent media were transferred to a fresh tube. Cells were washed with 10 mL of PBS solution by invert mixing and centrifuged again. The PBS wash was added to the spent media collected above, followed by the addition of Ni-NTA agarose and further IMAC purification for isolating the target peptide that may have been released into the medium due to cell death and lysis. The resultant cell pellet was resuspended in 10 mL of Pierce IP lysis buffer, and the suspension was sonicated for 2 min at 30% amplitude with a 5 s on/off cycle. The lysate was centrifuged at 3000*g* for 10 min, and the supernatant was subjected to IMAC for purification of the lanthipeptides from the intracellular environment. The purified peptides (acquired from cell lysate and spent medium combined) were buffer-exchanged to 50 mM Tris-HCl containing 100 mM NaCl at pH 8.0 (25 °C), followed by CylA digestion at 1:100 substrate/enzyme ratio (w/w) for 2 h at RT. The reaction was then quenched with 0.1% FA (final v/v), desalted using C18 ZipTips, and analyzed by MALDI-TOF MS and HR-MS/MS, as discussed in the sections below. For large scale preparation of CylL_L_″-S15T for quantitative antimicrobial activity determination, the acidified reaction was directly purified by HPLC (Vanquish Core HPLC system; Thermo) using a Kinetex 5 μm C18 100 Å, LC Column (250 × 10.0 mm; Phenomenex; part no.: 00G-4601-N0) as the solid phase. Solvent A (0.1% aqueous TFA) and solvent B (acetonitrile with 0.1% TFA) were used as the mobile phase. The mobile phase was maintained at 5% B for 5 min, followed by a gradient of 40–95% B over 15 min at a 2 mL/min flow rate. A final wash step of 95% B for 5 min was applied before equilibrating the column back to 5% B for 10 min to prepare for subsequent injections. CylL_L_″-S15T eluted between 13.5 and 14 min of the gradient, accounting for ca. 70–75% of B. The collected fractions were lyophilized and stored at −20 °C until further studies.

### Generation of an CylL_L_-S15T NDT Variant Library

A primer containing degenerate NDT codons at five amino acid positions along CylL_L_-S15T was used to create a mutant library. The following PCR conditions were used with Phusion polymerase in a 50 μL reaction volume aliquoted into 9 μL. Each reaction contained 1× Phusion HF buffer, 1 M betaine, 0.2 mM dNTPs, 0.125 μM of each primer, 0.5 μL polymerase, at least 10 ng of DNA template, and DI H_2_O added up to 50 μL. Initial denaturation was performed for 2 min at 95 °C, followed by 26 cycles of denaturation (20 s, 95 °C), annealing (30 s, 59 °C), and elongation (30 s, 72 °C), and a final elongation step (72 °C) for 5 min. Successful amplification was confirmed via gel electrophoresis. After amplification, 1 μL of Dpn1 enzyme was added to each 50 μL reaction and incubated at 37 °C for 1 h, followed by a Qiagen PCR cleanup per the manufacturer’s instructions. PCR linearized vectors and NDT library inserts were assembled via Gibson Assembly (GA) per the manufacturer’s instructions and subsequently dialyzed against DI H_2_O using a 0.02 μm membrane. ElectroMAX DH10B cells were transformed with the dialyzed GA reactions (2 reactions, 20 μL each) and recovered with 880 μL of Super Optimal broth with Catabolite repression (SOC) media for 1.5–2 h. Postrecovery, 100 μL of transformants was plated on Amp100 at 10^–3^ and 10^–4^ dilutions and incubated overnight at 37 °C. The remaining cells were used to inoculate 9 mL of LB (100 μg/mL Amp), incubated overnight at 37 °C, and the library DNA isolated using a Qiagen miniprep kit. NextGen sequencing was performed via SeqCenter, and the reads were analyzed using a custom Python script.

### High-Resolution Tandem Mass Spectrometry

The desalted protease-digested peptides were injected onto an Agilent 1290 LC–MS QToF instrument for HR-MS/MS analysis. LC separation was conducted at 50 °C on a 10–80% gradient of acetonitrile–water (+0.1% formic acid) over 11 min at 0.6 mL/min flow rate on a Phenomenex Aeris 2.6 μm PEPTIDE XB-C18 LC column (part no. 00F-4505-E0). Mass spectra were collected in positive mode at 10 spectra/s and 100 ms/spectrum. Tandem-MS fragmentation was achieved at normalized collision energies of 20, 25, and 30.

### Bioactivity Assay of Cytolysin and Haloduracin

Modified CylL_L_ and HalA2 were digested with CylA (described above) or AspN (NEB; 2 μg in LanM start buffer with 0.5 M imidazole incubated overnight at RT), respectively. Peptides were purified via aHPLC as described above and were resuspended in sterile, 1× PBS containing 20% MeOH (CylL_L_″) or DI H_2_O (Halβ). Halβ was produced with two amino acids (Ala-Gln) of its leader peptide remaining on its N-terminus (Figure 2B, presumably due to amino peptidase activity after AspN cleavage that removed three amino acids (see leader peptide sequence in Figure S3). The concentrations were then estimated using the Pierce Quantitative Colorimetric Peptide Assay according to the manufacturer’s protocol. Agar well diffusion assays were used to evaluate the antimicrobial activity of the modified peptide cores against *L. lactis* cremoris NZ9000 (CylL_L_″) or *L. lactis* CNRZ 117 (Halβ). A starter culture of the indicator strain was grown in M17 medium (Sigma) supplemented with 0.5% glucose postautoclaving (GM17 media) under aerobic conditions at 30 °C overnight. For the Halβ assay, agar plates were prepared by combining molten M17 agar (cooled to 42 °C) supplemented with 0.5% glucose and 300 μL of overnight bacterial culture to yield a final volume of 40 mL. The seeded agar was poured into a sterile Petri dish and allowed to solidify at RT, after which 100 pmol of modified peptides was spotted onto the agar plates. For CylL assays, an overnight culture of the indicator strain *L. lactis* cremoris NZ9000 was subcultured to OD 1. Base agar plates were prepared with GM17 containing 2% (w/v) agar. For soft agar overlay, 4 mL of GM17 containing 0.4% (w/v) agar at 42 °C was mixed with 20 μL of the subculture and uniformly poured over the base agar. Then 100 pmol of the test peptides (diluted in PBS) was spotted on the solidified soft agar overlay along with appropriate solvent controls (20% methanol in PBS). All plates were incubated at 30 °C overnight, and antimicrobial activity was qualitatively determined by the presence or absence of a zone of growth inhibition.

For IC_50_ determination, subcultured *L. lactis* cremoris NZ9000 at OD 1 was diluted to OD 0.1. The test peptides were serially diluted in 90 μL of GM17 media per well in a 96 well plate, followed by a 10 μL inoculation of the diluted culture in each well to reach a final OD of 0.01. The plates were incubated at 30 °C for 16 h before end point OD measurements (*T*_16_). OD_600_ of uninoculated GM17 media was also recorded as blank. 20% methanol in PBS (also serially diluted) was used as a solvent control (*T*_C_). The OD of the wells with untreated bacteria was also recorded and averaged (*T*_U_; *n* = 6). End point readings of the treated and untreated wells were subtracted from the blank and normalized against *T*_C_ (resulting in *T*_U–C_ and *T*_16-C_, respectively). The percentage of inhibition was calculated using the following formula; ((*T*_U–C_) – (*T*_16-C_)/*T*_U–C_) × 100)). The dose vs response nonlinear regression curve was plotted, and IC_50_ values were calculated using GraphPad Prism 10.

### Iodoacetamide (IAA) Reaction Conditions

Purified CylL_L_″-S15T was resuspended in 100 μL of DI H_2_O and sonicated in a water bath for at least 5 min. To a 50 μL reaction, 5 μL of 100 mM TCEP, 5 μL of 200 mM KH_2_PO_4_ buffer pH 7.5, and 30 μL of peptide were added. The reaction was incubated for 30 min at 37 °C to allow for the reduction of disulfide bonds, prior to the addition of 5 μL of 100 mM IAA (in KH_2_PO_4_, pH 7.5). Following IAA addition, the reaction was incubated for 30 min at 37 °C. Reactions were purified with C4 ZipTip and analyzed via MALDI-TOF MS. CylM-modified CylL_L_-S15T NDT mutants were purified by IMAC and subsequently buffer-exchanged into 50 mM Tris HCl pH 8 supplemented with 100 mM NaCl, followed by an overnight digestion with CylA at RT (100:1; substrate/enzyme). A portion of the digest was desalted using C18 ZipTips and analyzed by MALDI-TOF MS. The remaining digest was treated with IAA as described above, desalted using C18 ZipTips, and analyzed by MALDI-TOF MS.

### *N*-Ethylmaleimide (NEM) Assay

To a 50 μL reaction, 25 μL of DI H_2_O, 5 μL of 100 mM TCEP, 5 μL of 1 M citrate buffer (100 mM EDTA, pH 6), and 10 μL of peptide were added; it was assumed 100 μg of peptide was present when resuspended in DI H_2_O to a concentration of 100 μM. The reaction was incubated for 10–30 min at 37 °C to allow for the reduction of disulfide bonds, prior to the addition of 5 μL of 100 mM NEM (dissolved in ethanol). Following NEM addition, the reaction was incubated for 30 min at 37 °C. Reactions were purified with C4 ZipTip and analyzed via MALDI-TOF MS.

### Marfey’s Analysis

WT CylL_L_ was purified after expression in *E. coli,* as described previously.^38^ Commercial nisin from *L. lactis* (Sigma N5764-1G) containing 5% nisin (w/w) was further purified by HPLC.^69^ The absolute stereochemistry of the Lan cross-links was determined as previously described, with slight modifications.^69^ Briefly, 50 μg of dried peptides was subjected to hydrolysis by resuspending in 0.8 mL of 6 M DCl in D_2_O in a 4 mL amber glass vial, followed by sparging with a N_2_ stream for 30 s. The mixture was heated at 120 °C for 16 h with stirring. The resulting product was dried under vacuum in a speedvac followed by resuspension in 1 mL of deionized water and dried again. The drying step was repeated twice to remove any remaining DCl. Next, 0.6 mL of 0.8 M NaHCO_3_ (in H_2_O) and 0.4 mL of a 3 mg/mL solution of *N*^α^-(5-fluoro-2,4-dinitrophenyl)-l-leucinamide (L-FDLA; Sigma) in acetonitrile were added, followed by stirring in the dark (amber glass vial) at 67 °C for 3 h. Postderivatization, 100 μL of 6 M HCl was added, and the mixture was vortexed. The mixture was lyophilized and subsequently resuspended in 200 μL of acetonitrile by sonication. The suspension was centrifuged at 12,000*g* for 10 min, and the supernatant was analyzed by LC–MS on an Agilent 6545 LC/Q-TOF instrument. Chromatographic separation was obtained on a Kinetex 1.7 μm F5 100 Å, LC column (100 × 2.1 mm; Phenomenex; part no.: 00D-4722-AN). A column oven was maintained at 45 °C, and the mobile phase used was A: water with 0.1% formic acid and B: acetonitrile (no acid). At a constant flow rate of 0.4 mL/min, a gradient of 2–30% B over 2.5 min, 30–80% B over the next 7.5 min was maintained, followed by a wash step of 90% B for 5 min and a postrun equilibration stage of 2% B for 5 min. MS spectra were collected in the negative ion polarity mode. 0.5–1 μL injections were performed for each sample.

### Confocal Microscopy

For nuclear localization experiments, all steps were performed at RT. HEK293 cells were grown on poly-d-lysine-coated coverslips (Neuvitro 18 mm #1 thick) in 6-well plates and transfected with the appropriate constructs as mentioned in the sections above using lipofectamine 3000 (Invitrogen) as per the manufacturer’s instructions. Then, 48 h post-transfection, cell media were removed, and the cells were washed twice with 1 mL of 1× PBS. Cells were fixed in 4% paraformaldehyde for 10 min and washed three times with 1 mL 1× PBS each time. Cells were then permeabilized with 0.5% Triton X-100 (in 1× PBS) for 10 min and washed three times with 1 mL 1× PBS each time. Cells were blocked with 2% bovine serum albumin (BSA) for 1 h and washed two-three times with 1× PBS. Primary anti-FLAG antibody (8146T, Cell Signaling Technologies) diluted 1:1000 in 1× PBS-T was added (i.e., PBS supplemented with 0.1% Tween 20), and the sample was incubated for 3 h. After primary antibody removal and washing (3× with 1 mL 1× PBS), goat antimouse-Fluor647 secondary antibody (A-21235, Thermo Fisher) was diluted 1:1000 in 1× PBS-T and incubated for 1 h. Following another wash step (3× with 500 μL 1× PBS), the coverslips were placed onto a slide containing ∼7 μL of mounting media containing DAPI (Vector Laboratories, cat no. H-1200-10). The coverslip was allowed to cure for 30 min, and the edges were sealed onto the slide with clear nail polish. Slides were stored at −20 °C until visualization. Immunofluorescence was visualized using a Zeiss LSM880 microscope with 63× magnification and immersion oil (Immersol 518F). Images were analyzed using Zen software (2.3 SP1).

For ERL and PML experiments, HEK293 cells were grown in DMEM supplemented with 10% FBS (Gibco), 1× l-glutamine, and 1× MEM nonessential amino acids at 37 °C and 5% CO_2_. HEK293 cells (>95% viability) were grown on sterile poly-d-lysine-coated coverslips (Neuvitro 18 mm #1 thick) in 12-well plates at 0.5 × 10^5^ cell density. Following 24 h of cell attachment and growth, the cells were transfected with the appropriate endotoxin-free plasmids using Lipofectamine LTX with PLUS Reagent (Invitrogen) as per the manufacturer’s instructions at the highest lipofectamine concentration recommended. However, certain changes were made to the recommended protocol. Briefly, for each well, 5 μL of Lipofectamine LTX reagent was diluted in 50 μL of Opti-MEM reduced serum media and incubated for 5 min at RT. Simultaneously, 250 μL of Opti-MEM was used to dilute 5 μg of endotoxin-free DNA, and 5 μL of the PLUS reagent was followed by incubation for 5 min at RT. Thereafter, 50 μL of diluted DNA-mix was added to 50 μL of the diluted Lipofectamine LTX reagent and further incubated for 20 min at RT for complexation. Post incubation, 100 μL of the mix was added to the cells dropwise in different parts of the well. Cells were incubated at 37 °C for 48 h before immunostaining. No media change was done after transfection.

Cell media were removed 48 h post-transfection, and the cells were washed twice with 1 mL of 1× PBS containing 0.9 mM calcium chloride and 0.5 mM magnesium chloride (PBS-Ca/Mg). Cells were then fixed in 500 μL of 4% paraformaldehyde in PBS (Pierce) for 10 min and washed thrice with 1 mL 1× PBS-Ca/Mg. Cells were permeabilized with 0.1% Triton X-100 (in 1× PBS-Ca/Mg) for 10 min and washed three times with 1 mL 1× PBS-Ca/Mg each time. At this point, the protocols diverged for the ERL and PML experiments. For ERL experiments, all washes, blocking, and antibody solutions were made in PBS-Ca/Mg supplemented with 0.1% Tween-20 (PBST-Ca/Mg), whereas for PML experiments, no detergent was used hereafter, just PBS-Ca/Mg. Cells were blocked with a 0.45 μm-filtered blocking solution in the buffer consisting of 2% BSA and 22.52 mg/mL glycine for 1 h, followed by washing twice with the appropriate buffer.

For the ERL multiplexing experiment, the anti-FLAG antibody raised in mouse (8146T, Cell Signaling Technologies) and the recombinant anti-GRP94 antibody EPR22847-50 raised in rabbits (ab238126) were used as primary antibodies as 1:1000 dilutions in PBST-Ca/Mg supplemented with 2% normal goat serum. Cells were incubated with the primary antibodies overnight at 4 °C. After primary antibody removal and washing (thrice with 1 mL of 1× PBST-Ca/Mg for at least 5 min each time), goat antimouse-Fluor647 secondary antibody (A-21235, Thermo Fisher) and goat antirabbit IgG H&L (Alexa Fluor 555) preadsorbed (ab150086) diluted 1:1000 in 1× PBST-Ca/Mg were added and incubated for 1 h. Following another wash step (thrice with 500 μL of 1× PBST-Ca/Mg, at least 5 min each time), the coverslips were placed onto a slide containing ∼20 μL of VECTASHIELD Vibrance Antifade Mounting Medium with DAPI (H-1800). The coverslip was allowed to cure for 24 h at RT before visualization. Immunofluorescence was visualized using a Zeiss LSM900 microscope with 63× magnification and immersion oil (Immersol 518F). Images were analyzed using ZEISS ZEN software v3.9.

For the PML experiment, the anti-FLAG antibody raised in mouse (8146T, Cell Signaling Technologies) was used as the primary antibody in 1:1000 dilution in PBS-Ca/Mg supplemented with 2% normal goat serum and incubated overnight at 4 °C. After primary antibody removal and washing (thrice with 1 mL of 1× PBS-Ca/Mg for at least 5 min each time), goat antimouse-Fluor647 secondary antibody (A-21235, Thermo Fisher) diluted 1:1000 in 1× PBS-Ca/Mg and supplemented with DAPI (1 μg/mL) was added and incubated for 1 h. Cells were washed thrice with 500 μL of 1× PBST-Ca/Mg at least 5 min each time. For PM counterstaining, cells were incubated in the dark for 15 min with 500 μL of CellBrite Orange cytoplasmic membrane dye (Cat #30022; Biotium) at a 5 μL/mL dilution following the manufacturer’s protocol. Cells were then washed with PBS thrice, and the coverslips were placed onto a slide containing ∼10 μL of 1× PBS (no Ca/Mg), sealed with clear nail polish, and immediately analyzed under the microscope. Immunofluorescence was visualized using a Zeiss LSM900 microscope with 63× magnification and immersion oil (Immersol 518F). Images were analyzed using ZEISS ZEN software v3.9.

## Supporting Information Available

Supporting Information Figures S1–S11 and nucleotide sequences used (PDF)
