# Supplementary material for: Expression and Subcellular Localization of Lanthipeptides in Human Cells
Source: ACS Synth Biol. 2024 Jun 26;13(7):2128–40. doi: 10.1021/acssynbio.4c00178 (PMC11264318; doi:10.1021/acssynbio.4c00178)
Supplement: Supplementary file 1 — sb4c00178_si_001.pdf [file sb4c00178_si_001.pdf]

Supporting Information

Expression and Subcellular Localization of Lanthipeptides in Human Cells

Sara M. Eslami[1,§], Chandrashekhar Padhi[1,§], Imran R. Rahman[2], and Wilfred A. van der Donk[1,2,*]

[1]Department of Chemistry and Howard Hughes Medical Institute, University of Illinois at Urbana-Champaign, Urbana, Illinois 61801, USA

[2]Department of Biochemistry, University of Illinois at Urbana-Champaign, Urbana, Illinois 61801, USA

§ These authors contributed equally to this study.

* E-mail vddonk@illinois.edu , 217 244 5360

All data in the main text figures and Supporting Information Figures can be found at: van der Donk, Wilfred; Eslami, Sara; Padhi, Chandrashekhar (2024), “Data associated with Expression of Lanthipeptides in Human Cells”, Mendeley Data, V1, doi: 10.17632/j8fch2ch89.1

Primers and codon optimized genes used in this study

SME_CylLL-S15T.F1	CTGCGTCAACGGCTGCATGCGGCTGGGTGGGCGGCG
SME_CylLL-S15T.R1	GCATGCAGCCGTTGACGCAGCAGCAGTAGCAGCCACG
SME_pCMV_NLS.R2	cactttgcgtttcttcttTGGGCCCGGATTTTCTTCG
SME_pCMV_NLS.F2	aagaagaaacgcaaagtgGACTACAAGGACGACGACGAC
SME_NLS_pCMV-FLAG	CGAAGAAAATCCGGGCCCAaagaagaaacgcaaagtgGACTACAAG
SME_CylLL_NDT.R3	ccgtgactccagtgaagattccAHNgcccacccaAHNgcatgcagccgttgacgcagcAHNagtagcagcAHNggcgcaAHNtggagtcgtc
CP_ER_CylM_FP	gccagcggccgctgccaagcttccgagctctcgaattcatggagcagaagttgatatccgaggaagatctgggctca
CP_CylLL-ERL-RP	gctgaaggtacgctgtatctcagtcagtcaagctagctcacaattcgtctttgccagcgccacagtgcttgagggacaccactaccgtgactcca
CP_PML_CylLL_FP	gccagcggccgctgccaagcttccgagctctcgaattcatgggatgtattaaatcaaaaaggaaagacggggactacaaggacgacgacgacaaggaa
CP_PML_CylLL_P2A-hang_RP	tgggcccggattttcttcgacatcacccgcttgtttgagcaaggaaaagttcgtggcgccagcgccacagtgcttgagggacaccactaccgtgac
CP_PML_CylM_FP	gctcaaacaagcgggtgatgtcgaagaaaatccgggcccagagcagaagttgatatccgaggaagatctgggctca
CP_PML_CylM_RP	gtctgtgagctgaaggtacgctgtatctcagtcagtcaagctagctcagagctcgaaaagcaaggcgttagggatct
H. sapiens codon-optimized CylM	tcagaggacaacctcatcaacgttctttcaatcaacgagagatgtttccttttgaagcaatctgggaaggagaagtacgacataaagaacttgcaagcttggaaggagagaaagagtgtgcttaagcaagacgacttggactaccttatcaagtacaagtacgagtctttggacaacttcggacttgggatcacacccatcgagaacttccctgacaaggaagtcgcaatccaatacatcaaggaccaatcctggtacatattcttcgagtcaatact

	cgactcatacaacgactctgaggagaagctcctcgaggtggacgcttcttaccctttccgctacttcttgcaatacgctcgattgttcttgctcga<br>cctcaactccgagctcaacatctgtactaaagagttcatcataaacctcttggagactctcactcaagagttgatacaccttacatccaagact<br>cttgtccttgacttgcacacgttcaagaagaacgagccactcaaggggaacgacagttccaagagattcatctactacctcaagaagcgatt<br>caactccaagaaggacatcatcgctttctacacgtgttaccccgagctcatgcggatcacagtggtgcggatgagatactttcttgacaatac<br>gaagcagatgctcatccgggtcactgaggacttgccttccatccaaaactgtttcaacatccaatccagcgagctcaacagtatcagtgagtc<br>tcaaggggactcacactccaggggaaagacagtgtccacgctcactttctcagacggcaagaagatcgtctacaagcctaagataaactc<br>cgagaacaagctcagagacttcttcgagttcttgaacaaggagctcgaggcagacatctacatcgttaagaaggttacacgtaacacttactt<br>ctacgaggagtacatagacaatatcgagattaacaatatagaagaggtcaagaagtactatgagcggtacgggaagttgatcggtatcgc<br>gttcctcttcaacgtcactgatctccactacgagaatattatcgcgcacggggagtaccctgtcatcatcgacaacgagactttcttccaacaa<br>aacatacccatcgagttcggcaactctgccactgtcgacgctaagtataagtacttggacagtatcatggtaactgggctcgtgccctaccttg<br>ccatgaaggacaagtccgacagtaaggacgagggtgtgaacctctctgctcttaacttcaaggagcaaagtgtccccttcaagatattgaag<br>atcaagaacactttcactgacgagatgaggttcgagtaccaaacacacatcatggacacagctaagaacacacctataatgaacaacga<br>gaagatttcattcatttcatacgagaagtacatcgttacaggtatgaagtccatcctcatgaaggctaaggactctaaaaaaaagatcctcgc<br>atacatcaacaacaatctccaaaacttgatcgtaagaaacgtcatccgccccacacaacggtacgcggacatgctcgagttcagttaccac<br>ccaaactgtttctctaacgcaatcgagcgggagaaggtccttcacaacatgtgggcttacccttacaagaacaagaaggttgtccactacga<br>gttctctgaccttatagacggagacatccccatcttctacaacaacatctcaaagacatccctcatcgcttccgacggatgcttggtcgaggac<br>ttctaccaagagagcgcattgaaccgatgtttgaacaagataaacgacctttgcgacgaggacatcagtatccaaacagtttggctcgagat<br>agctctcaacatctacaatccatataagtacataaacgaccttaagaatcaaaactccaataagtacatctacactgggctcgagctcaacg<br>gaaagatcatacaagcttgtcaaaagatcgagaagaagattttcaagcgggcaattttcaataagaagacgaacactgtaaactggatcg<br>acataaagcttgaccaagactggaacgttggaatcctcaacaacaacatgtacgacggtctcccaggcatattcatcttctacgtcgcactca<br>agtacatcacaaagaatcacaagtacgactacgtcatagagtgtatcaagaactcaatatacactatcccttccgaggacatcctctccgcct<br>tcttcgggaaggggtccttgatatacccattgttggtggactaccgactcaacaacgacataaacagtctcaacgttgcagtcgagatcgctg<br>acatgctcatagagaagaagcctataaacaacggggagcttaagaacgactggatccacggacacaactccatcatcaaggtcctgcttc<br>ttttgtccgagatcactgaggacgagaagtacaggaagttctcactcgagatcttcgagaagctctccgaggagccctacttcaacttcaggg<br>gcttcggacacgggatctactcatacgtccacctcttgtccaagttcaacaggatcgacaaggctaactccttgcttcacaagatcaaggagt<br>catacttcgaagaggagccaaagaacaactcttggtgcaagggaactgtgggcgagctcttggctacgatcgagttgtacgacgacaaca<br>tatccaatattgacatcaataagactatagcgtacaagaacaaggactgtctttgtcacggaaacgctggaacactcgagggcttgatacaa<br>ctcgctaagaaggaccctgagacttaccaatacaagaagaacaagctcatatcatacatgctcaacgacttcgagaagaacaacacact<br>caaggtagcggggagtgagtacttggagtcacttggattcttcgtgggaatctccggagtcggatacgagttgctcagaaaccttgactccga<br>gatccctaacgccttgcttttcgagctc
H. sapiens codon-optimized CylLL-S15T	tcacaagaccctaactctgagaacctcagtgtagtgccctctttcgaggagctttctgttgaggagatggaagccattcaaggaagtggggat<br>gtgcaagctgagacgactccagtgtgcgccgtggctgctactgctgctgcgtcaacggctgcatgcggctgggtgggcggcggaatcttcac<br>tggagtcacggtagtggtgtccctcaagcactgttga
H. sapiens codon-optimized HalM	aaaaccccactgacgtcagagcatccttcagtccctacgactctgcctcacactaatgacaccgattggctcgaacaacttcatgacatactc<br>tccatccccgtgactgaggaaatccagaaatattttcacgcagagaacgatctcttctcattcttctataccccctttcctgcaattcacatatcaga<br>gtatgagcgactactttatgacctttaaaaccgatatggccctgattgaacgacaatctcttctccaatctactttaactgctgtacatcatcggct<br>gttccaccttacacatcgcacgctgatctcagagatgcacattgataaacttacagtgggtcttaacggtagcacccctcacgaaaggtatat<br>ggattttaatcacaaattcaacaaaacatccaaatctaagaatctcttcaacatatacccgatactgggcaagctggtagtcaacgagactct<br>ccgaacgataaattttgtaaagaaaattatacaacactatatgaaggactatctcttgctcagtgacttcttcaaggagaaagacctgagactg<br>accaaccttcaactgggagtcggcgacacccatgttaatggccaatgcgtgacaattctgacatttgcgagcggacaaaaggtagtgtataa<br>gccgcgaagtctgtctatcgataagcaatttggcgaatttatagagtgggtaaattcaaagggctttcaaccttctcttcgcattccaatagccatt<br>gacaggcaaacctacggatggtacgagtttataccccatcaagaagctacaagcgaggacgaaattgagcgctattactcccgcataggt<br>ggatacctcgcaattgcttatctcttcggggctacggatctgcacctggataacctcatcgcgtgtggagaacaccccatgctgattgatctcga<br>aaccctcttcactaatgacctggactgctatgattccgcctttccatttccagccttggctcgcgagctcacgcaatcagtattcggcaccctcat<br>gttacccatcactatcgcatctggtaagttattggacattgacctttcagccgtcggcggcggaaaaggcgtccaaagcgagaagattaaaa<br>cttgggtcatcgttaatcaaaagacggatgagatgaaattggttgaacaaccctatgtgactgagagcagccagaataaacccacggtcaa<br>cggcaaggaagcaaatataggcaattacattccacatgtgaccgacgggtttcgcaaaatgtaccgattatttcttaatgaaatcgatgaact<br>gatggatcataatgggcctattttcgcattcgagagctgtcaaatccggcacgtgtttagggctactcatgtatatgccaaatttctggaagcttct<br>actcatcctgattacttacaagaaccaactaggcgcaataaactctttgaaagcttctggaatataacatccctcatggcgccttttaagaaaat<br>tgttccacacgagatcgcagaactcgagaatcatgatattccatatttcgttttgacatgtggcggcaccatcgttaaggatggatacggccga<br>gacatagcagatctgttccaatctagttgtatagaaagggtgactcatagacttcagcaactgggatccgaagacgaggcccgacaaatca<br>gatacattaagtccagtctggcaaccctgaccaacggggactggaccccatcacacgaaaagaccccgatgagtccagctagcgcgga<br>ccgcgaagacgggtattttctccgcgaagcccaagcgattggagatgacattcttgctcaactcatttgggaagatgacaggcacgctgcat<br>acctgatcggtgtctcagtcggcatgaatgaggcggtgaccgtatcccctctgactccaggaatctatgatggtacactggggatcgtgctctt<br>ctttgatcaactcgcgcaacaaacgggtgagacgcattatagacatgctgcagacgcattattggaaggcatgttcaaacaattgaagcca<br>gagttgatgccctcaagcgcatacttcggacttgggagtcttttctacggtcttatggtgctcggtctgcaacgatccgacagccacattataca<br>gaaggcctatgaatatcttaagcacttggaagaatgtgtgcagcatgaagagacgcccgatttcgtgtcaggccttagtggcgtgctctatatg<br>cttaccaaaatatatcagttgactaatgagccacgcgtatttgaggtagccaaaaccacagcttcccgacttagtgtcttattagatagcaaac<br>aacccgacaccgtcttgacaggcttgagtcacggcgccgccggttttgccctcgcgctcctcacctatggtactgccgctaacgacgaacaa<br>ctcctcaaacaagggcactcctatctcgtatatgagcgaaatagatttaataagcaagagaataattgggttgaccttcggaaaggaaatgc<br>ctatcaaacattctggtgtcatggtgcccctggtattggaattagcaggctgttactggctcaattttatgatgacgagttgttgcacgaggaactt<br>aacgccgctctgaataagactataagcgatggttttgggcacaaccattctctgtgccacggagatttcggaaacctcgatctgctgttgctcta<br>cgcccaatatacaaataatccagaaccaaaagaactggccaggaagctggccatatcctctatagatcaggctcatacctatggctggaa<br>actcggcctgaatcacagtgaccaattacaaggaatgatgctcggcgtgacaggaataggataccagttacttagacatataaaccctacg<br>gttccctctatattggccctggaactccccagtagtacactgaccgaaaaggaactcagaatccacgaccgggca
H. sapiens codon-optimized HalA2	gttaattctaaagatctccgaaatcctgaatttcggaaagctcagggcctgcaattcgtcgacgaggttaacgagaaagaattgtcaagcctg<br>gccgggtctggagatgtacatgcgcaaacgacatggccctgtgcaacggtcggagtcagcgtcgcgttatgcccaaccactaagtgcacct<br>ctcagtgctag

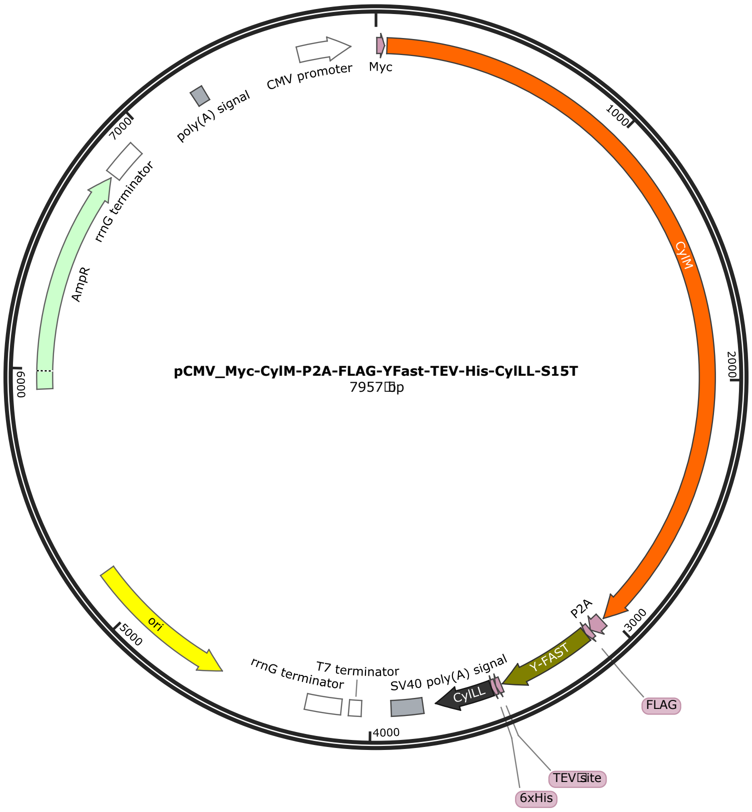

Figure S1: Mammalian expression vector for lanthipeptide production.

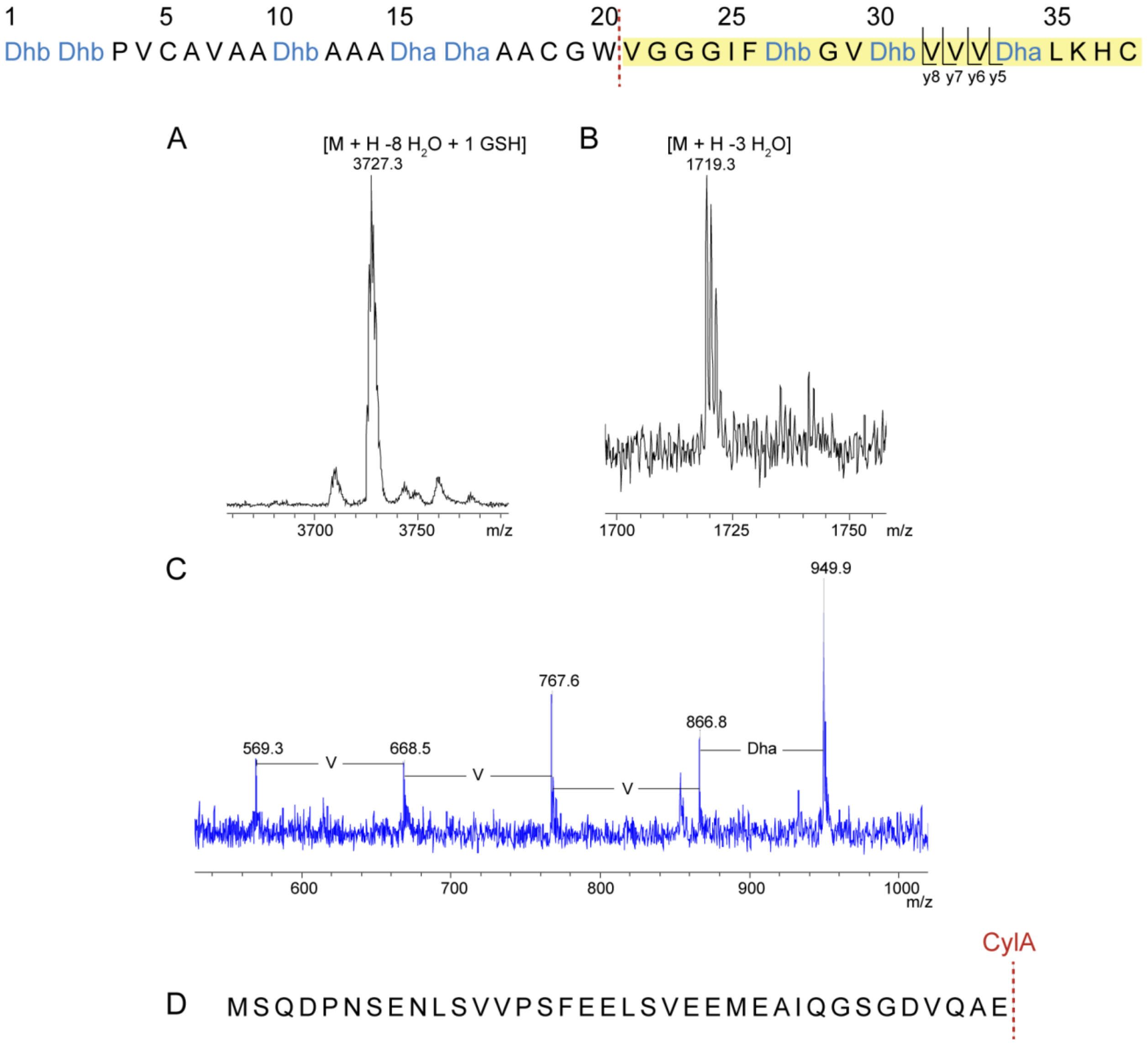

Figure S2: Presence of glutathione adduct in CylL$_L$ co-expressed with CylM in HEK293 cells and digested with CylA. (A) MALDI-TOF mass spectrum of 8-fold dehydrated CylL$_L$" with a GSH adduct. The peptide was purified via Ni-NTA affinity chromatography and digested with CylA. Residue numbering used in this study is indicated. (B) MALDI-TOF mass spectrum of the yellow highlighted CylL$_L$ fragment post-chymotrypsin digest. (C) LIFT analysis of the 1719 Da fragment. (D) Leader peptide sequence of CylL$_L$ with the CylA cleavage site indicated with an arrow. The modified core peptide sequence of CylL$_L$ is shown at the top of the figure.

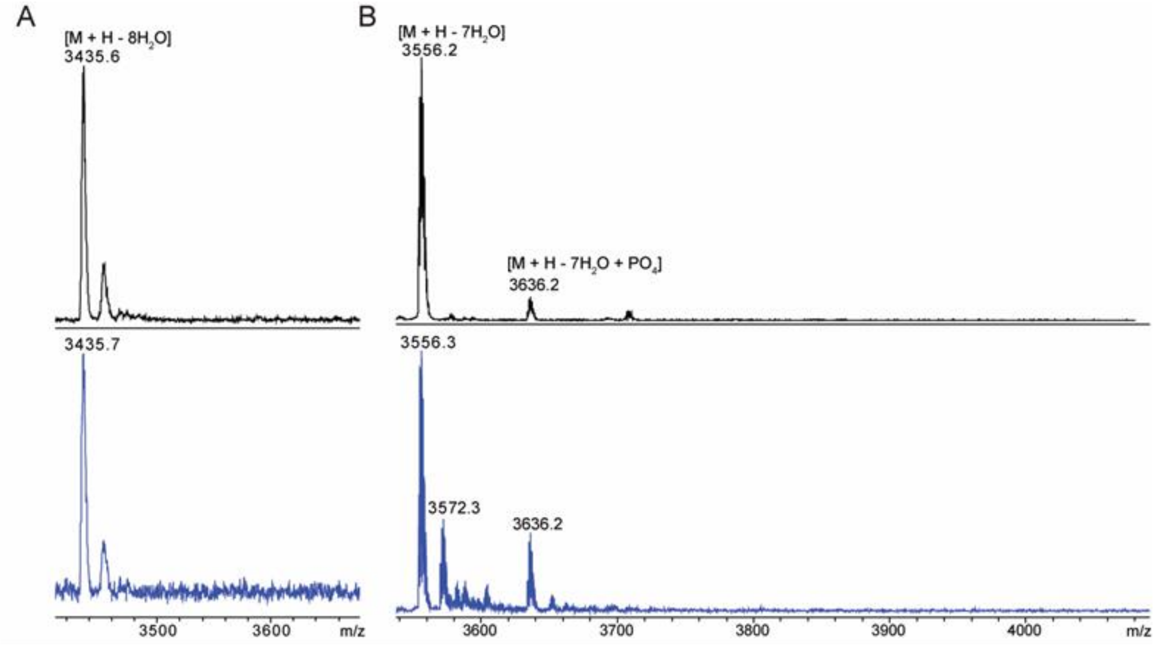

C MVNSKDLRNPEFRKAQGLQFVDEVNEKELSSLAGSGDVHAQ

D (residue numbers 1, 5, 10, 15, 20)
Dhb Dhb **WPCA** Dhb **VGV** Dha **VALCP** Dhb Dhb **KC** Dhb **SQC**

Figure S3: MALDI-TOF mass spectra of (A) $CylL_L$-S15T and (B) HalA2 co-expressed with CylM and HalM2, respectively, before (black) and after (blue) IAA (A) or NEM (B) reaction. Peptides were expressed in Expi293F cells and purified via Ni-NTA chromatography and analytical HPLC. Modified $CylL_L$-S15T and HalA2 were digested with CylA and GluC, respectively. Reaction products were desalted via C4 ZipTip. (C) Leader peptide sequence of HalA2. (D) Residue numbering of the modified core peptide sequence of HalA2 (7x dehydrated). Thioether rings are not shown for clarity.

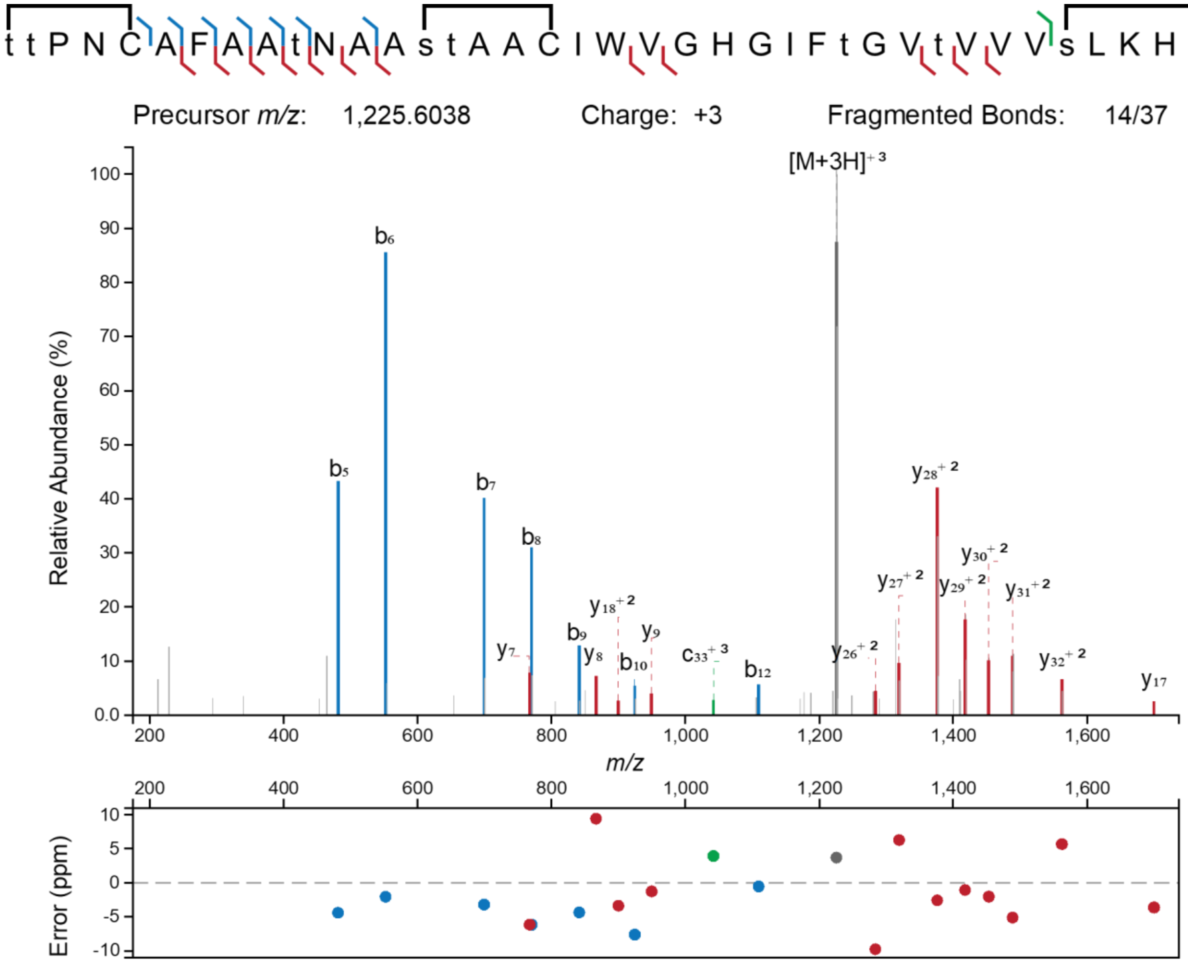

Figure S4: High-resolution MS/MS spectrum of CylA-digested CylL$_L$-S15T variant NDT1 (8x dehydrated product) co-expressed with CylM in Expi293F cells. Peptide was purified via Ni-NTA affinity chromatography prior to protease digestion. A graph of the ppm errors for each identified ion is shown.[1] The IAA assays shown in Figure 5 indicated that the vast majority of the peptide is cyclized. The amino acid sequence of the core peptide is shown on top with brackets marking the residues involved in the macrocycle formation. Residues in lower case represent the dehydrated amino acids as shown by the observed fragment ions.

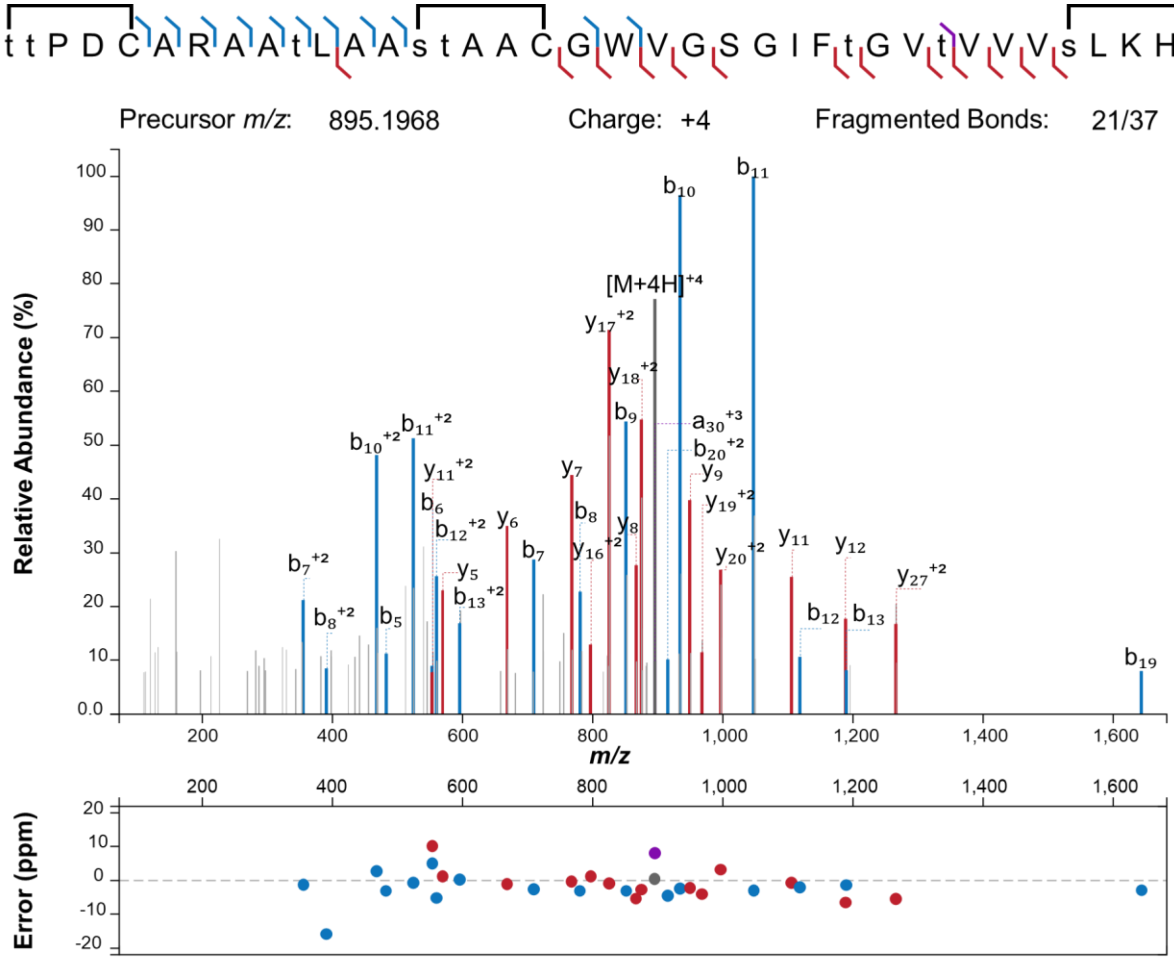

Figure S5: High-resolution MS/MS spectrum of CylA-digested CylL$_L$-S15T variant NDT2 (8x dehydrated product) co-expressed with CylM in Expi293F cells. Peptide was purified via Ni-NTA affinity chromatography prior to protease digestion. Fragmentation of the 8x dehydrated product is shown. Brackets represent the residues undergoing cyclization. A graph of the ppm errors for each identified ion is shown.[1] The IAA assays shown in Figure 5 indicate that the vast majority of the peptide is cyclized. The amino acid sequence of the core peptide is shown on top with brackets marking the residues involved in the macrocycle formation. Residues in lower case represent the dehydrated amino acids as indicated by the observed fragment ions.

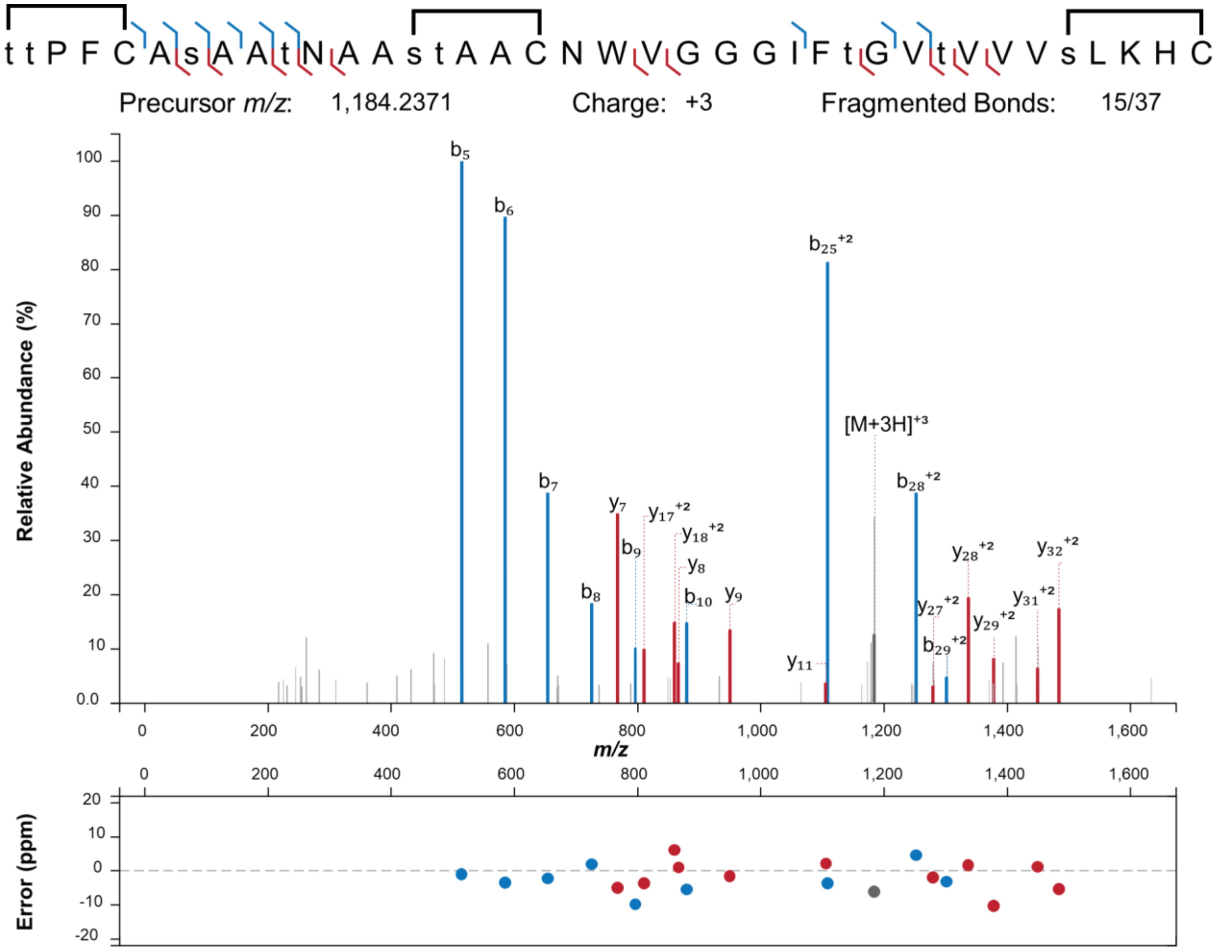

Figure S6: High-resolution MS/MS spectrum of CylA-digested CylL$_L$-S15T mutant NDT3 (9x dehydrated product) co-expressed with CylM in Expi293F cells. Peptide was purified via Ni-NTA affinity chromatography prior to protease digestion. Fragmentation of the 9x dehydrated product is shown. Brackets represent the residues undergoing cyclization. A graph of the ppm errors for each identified ion is shown.[1] The IAA assays shown in Figure 5 indicate that the vast majority of the peptide is cyclized. The amino acid sequence of the core peptide is shown on top with brackets marking the residues involved in the macrocycle formation. Residues in lower case represent the dehydrated amino acids as supported by the observed fragment ions.

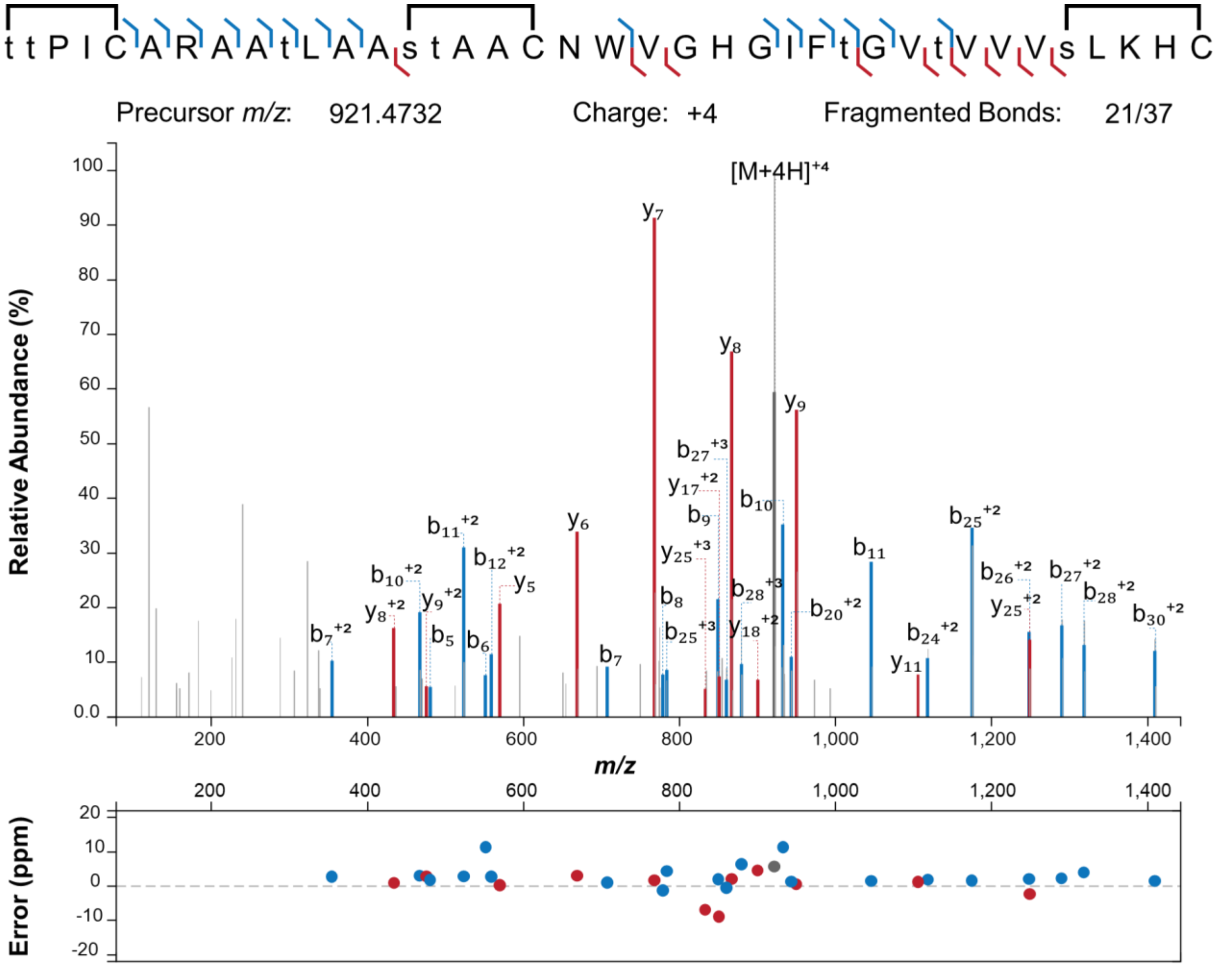

Figure S7: High-resolution MS/MS spectrum of CylA-digested CylL$_L$-S15T mutant NDT4 (8x dehydrated product) co-expressed with CylM in Expi293F cells. Peptide was purified via Ni-NTA affinity chromatography prior to protease digestion. Fragmentation of the 8x dehydrated product is shown. Brackets represent the residues undergoing cyclization. A graph of the ppm errors for each identified ion is shown.[1] The IAA assays shown in Figure 5 indicate that the vast majority of the peptide is cyclized. The amino acid sequence of the core peptide is shown on top with brackets marking the residues involved in the macrocycle formation. Residues in lower case represent the dehydrated amino acids.

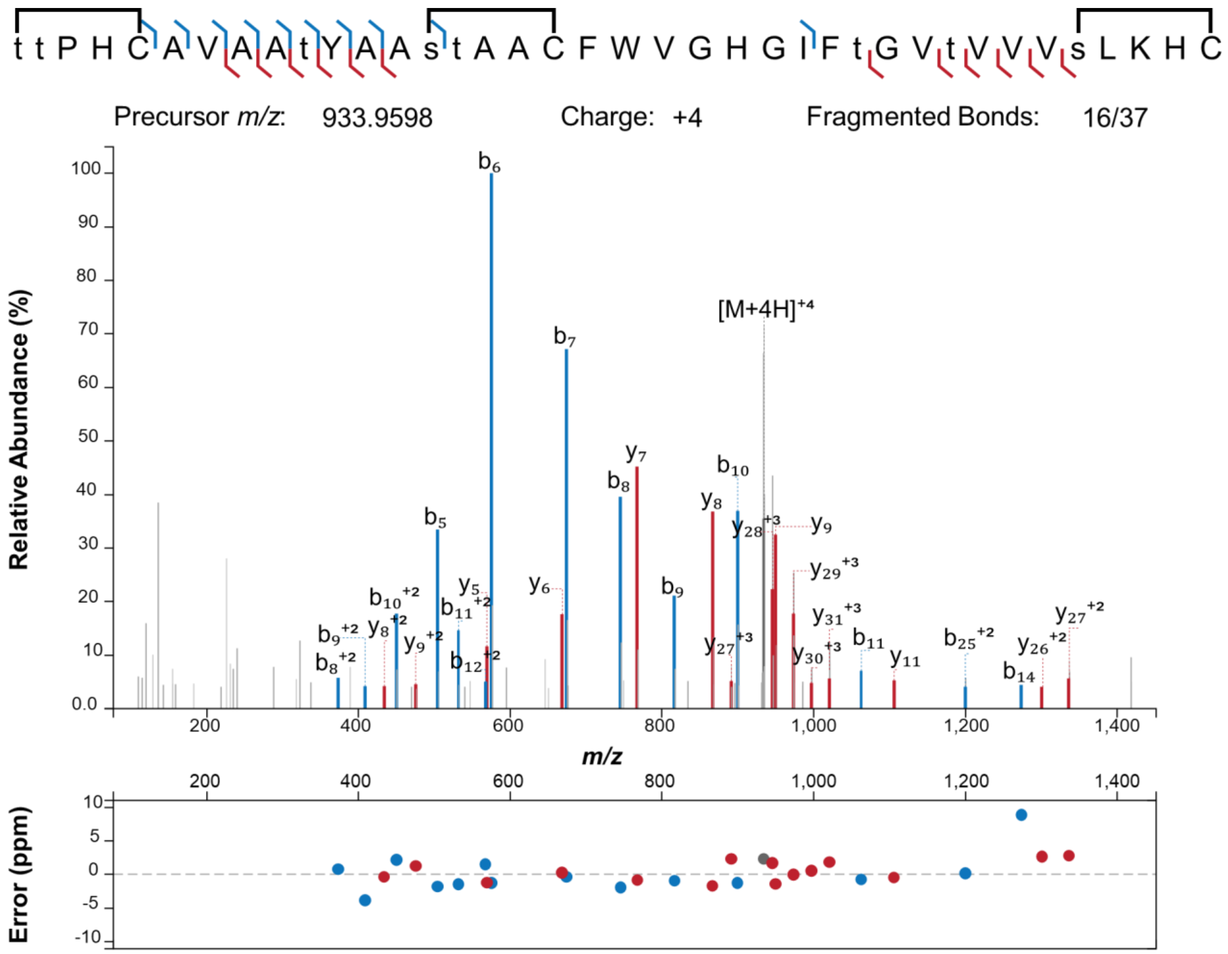

Figure S8: High-resolution MS/MS spectrum of CylA-digested CylL$_L$-S15T mutant NDT5 (8x dehydrated product) co-expressed with CylM in Expi293F cells. Peptide was purified via Ni-NTA affinity chromatography prior to protease digestion. Fragmentation of the 8x dehydrated product is shown. Brackets represent the residues undergoing cyclization. A graph of the ppm errors for each identified ion is shown.[1] The IAA assays shown in Figure 5 indicate that the vast majority of the peptide is cyclized. The amino acid sequence of the core peptide is shown on top with brackets marking the residues involved in the macrocycle formation. Residues in lower case represent the dehydrated amino acids.

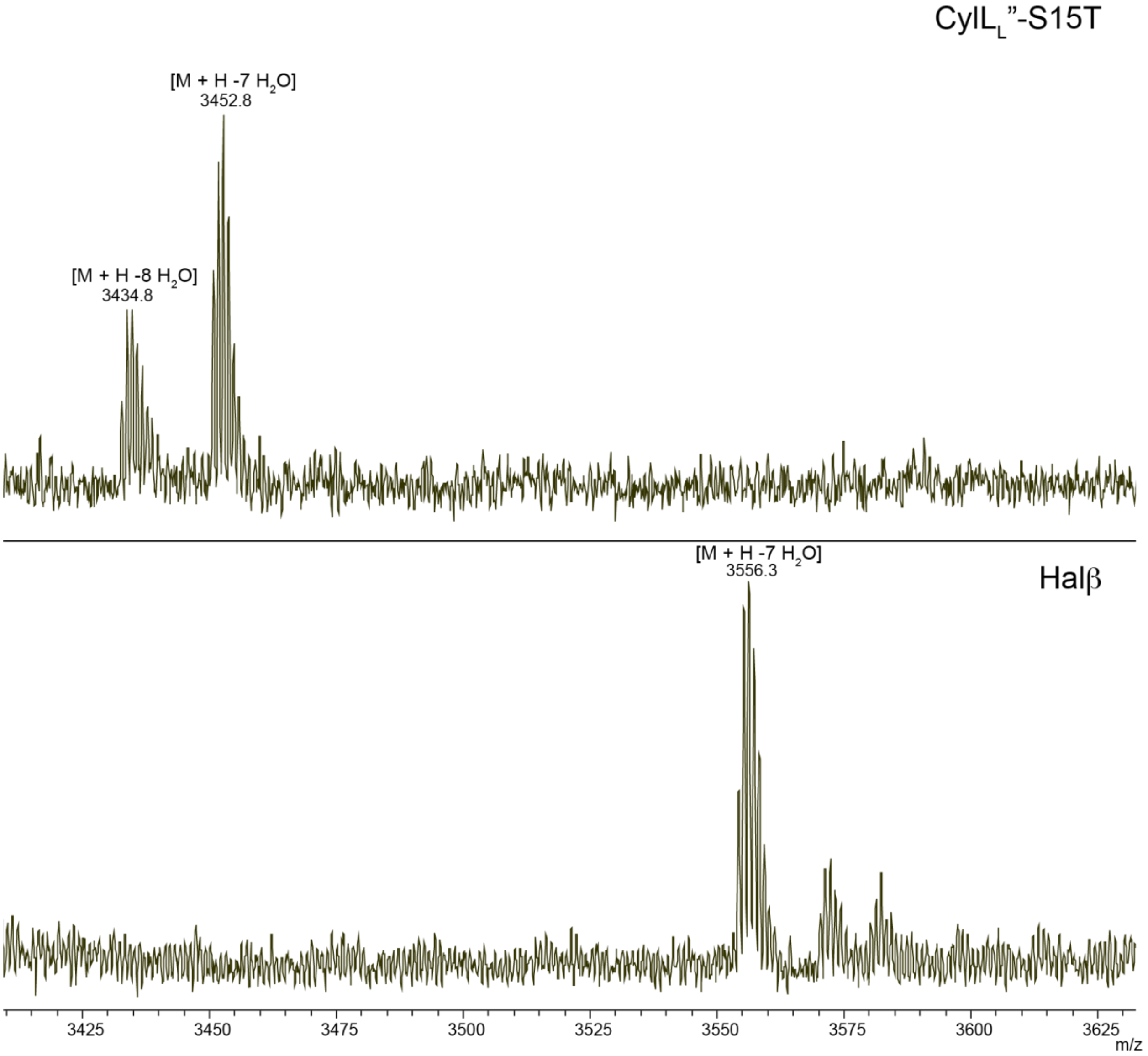

Figure S9: MALDI-TOF mass spectra of nuclear targeted CylL$_L$-S15T and nuclear targeted HalA2 co-expressed with CylM and HalM2, respectively, in Expi293F cells. CylL$_L$-S15T and HalA2 were digested with CylA and GluC, respectively. The GluC digest leaves 13 amino acids originating from the leader peptide on the N-terminus of Halβ (see sequence in Figure S3C).

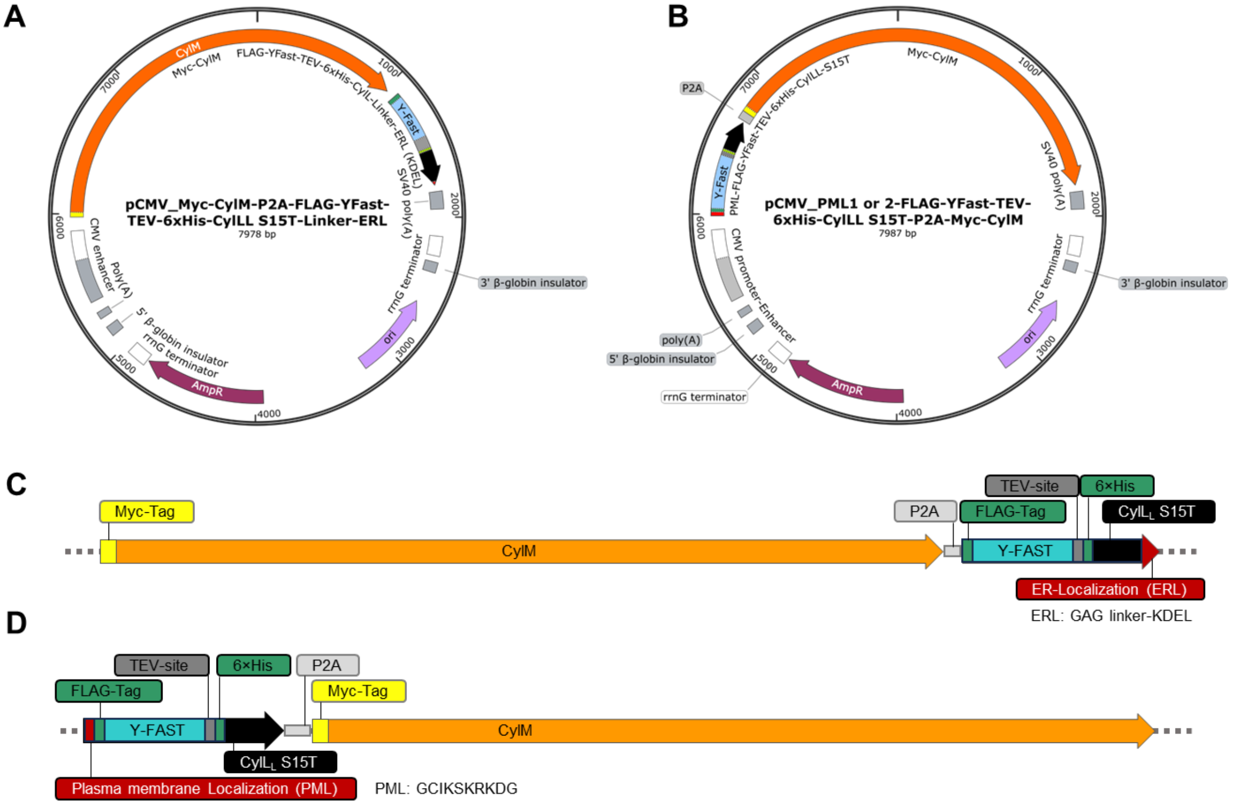

Figure S10: ER and PM localization vector maps. (A) Schematic representation of the plasmid vector used for expression of CylL$_L$-S15T fused with a C-terminal KDEL signal for endoplasmic reticulum localization (ERL). (B) Vector map schematics for CylL$_L$-S15T signaled to the plasma membrane (PML). (C) The polycistronic architecture for ERL consisted of the CylM maturase followed by the ribosomal skipping and self-cleaving P2A site, further extended by a fused FLAG tag, Y-FAST tag, TEV cleavage site, hexa-His tag, the peptide target CylL$_L$-S15T, a GAG-linker and the ERL-signal KDEL. (D) In case of PML, the vector architecture was refactored for a similarly fused CylL$_L$-S15T to be expressed along with the maturase CylM. The PML tag implemented in this study (GCIKSKRKDG)[2] was annexed to the N-terminus of the fusion peptide.

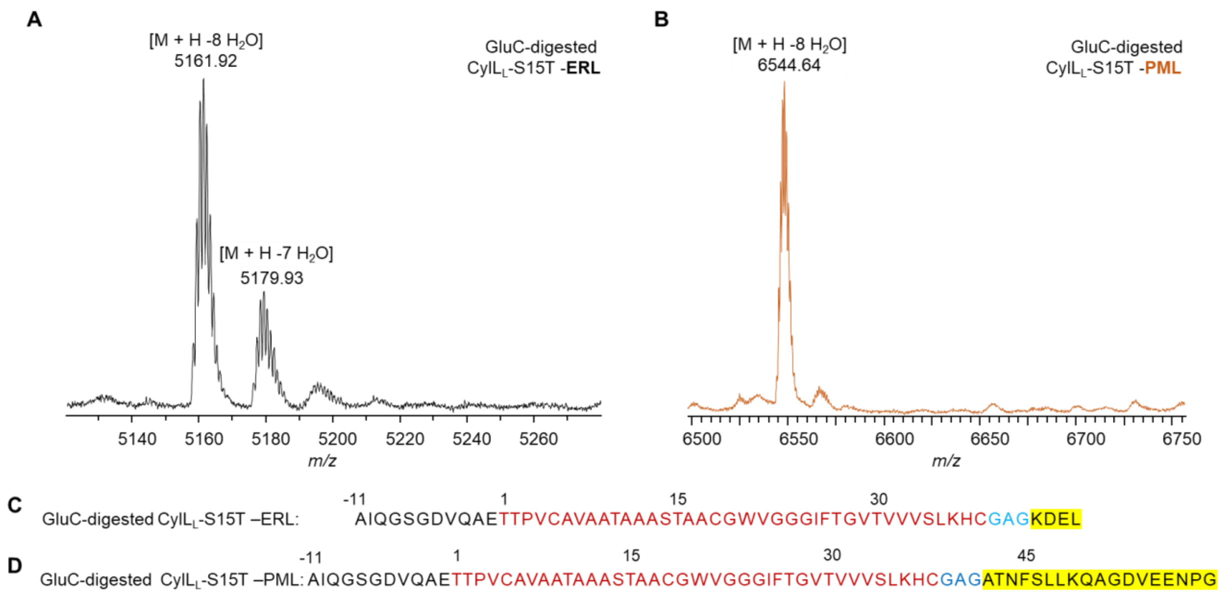

Figure S11: MALDI-TOF mass spectra of endoplasmic reticulum and plasma membrane targeted CylLL-S15T co-expressed with CylM in Expi293F cells. (A) GluC-digested CylLL-S15T shows up to eight dehydrations even with the C-terminus extended with the signal for localization to the endoplasmic reticulum (ERL; black spectrum; sequence in panel C). (B) GluC-digested CylLL-S15T showing up to eight dehydrations (PML; orange spectrum). This peptide contains a part of the P2A site (see panel D) (C) The ultimate residue of CylLL-S15T (Cys participating the ring C) is followed by a GAG linker (blue) preceding the ERL signal-KDEL (highlighted in yellow). Core peptide residues are in red. Remnant residues of the leader peptide after GluC digestion are shown in black. (D) According to the architecture of our PML expression vector, the P2A-based ribosomal skipping and self-cleavage results in the C-terminus of CylLL-S15T peptide extended by twenty-one amino acids (residues highlighted in yellow). This extension did not attenuate CylM activity in recognizing and successfully dehydrating the peptide with up to eight water losses as observed by MALDI-TOF MS. GAG-linker between the core and the P2A site is shown in blue. Core peptide residues are in red. Remnant residues of the leader peptide after GluC digestion are shown in black. The numbering of amino acids starts at the first residue of the core peptide.

REFERENCES

1. Brademan, D. R.; Riley, N. M.; Kwiecien, N. W.; Coon, J. J., Interactive peptide spectral annotator: A versatile web-based tool for proteomic applications. *Mol. Cell. Proteom.* **2019**, *18* (8, Supplement 1), S193-S201.

2. Corbett-Nelson , E. F.; Mason , D.; Marshall , J. G.; Collette , Y.; Grinstein , S., Signaling-dependent immobilization of acylated proteins in the inner monolayer of the plasma membrane. *J. Cell Biol.* **2006**, *174* (2), 255-265.
